# Estimated health benefits, costs, and cost-effectiveness of implementing WHO's sodium benchmarks for packaged foods in India: a modelling study

**DOI:** 10.1016/S2468-2667(24)00221-4

**Published:** 2024-10-30

**Authors:** Kathy Trieu, Liping Huang, Leopold N Aminde, Linda Cobiac, Daisy H Coyle, Mary Njeri Wanjau, Sudhir Raj Thout, Bruce Neal, Jason H Y Wu, Lennert Veerman, Matti Marklund, Rachita Gupta

**Affiliations:** a The George Institute for Global Health, University of New South Wales, Sydney, NSW, Australia; b School of Medicine and Dentistry, Griffith University, Gold Coast, QLD, Australia; c The George Institute for Global Health, Hyderabad, India; d Department of Epidemiology and Biostatistics, Imperial College London, London, UK; e School of Population Health, University of New South Wales, Sydney, NSW, Australia; f Department of Epidemiology, Johns Hopkins Bloomberg School of Public Health, Baltimore, MD, USA; g The Welch Center for Prevention, Epidemiology and Clinical Research, Johns Hopkins University, Baltimore, MD, USA; h Department of Public Health and Caring Sciences, Uppsala University, Uppsala, Sweden; i WHO, Country Office India, New Delhi, India

## Abstract

**Background:**

Excess dietary sodium intake has been associated with death and disability. WHO has released global sodium benchmarks for packaged foods to support countries to reduce population sodium intake. This study aimed to assess the potential health effect, costs, and cost effectiveness of implementing these WHO sodium benchmarks in India.

**Methods:**

We used a multiple cohort, proportional multistate, life table (Markov) model to estimate the health gains and cost effectiveness for adults if sodium content in packaged foods complied with the WHO benchmarks compared to the status quo. We used India-specific dietary surveys, food composition tables, foods sales data, and sodium content data from packaged food labels to estimate sodium intake before and after the intervention. Data on blood pressure, cardiovascular disease, and chronic kidney disease burden were obtained from the Global Burden of Diseases, Injuries, and Risk Factors study, and the effect of sodium reduction on blood pressure and disease risk was modelled on the basis of meta-analyses of randomised trials and cohort studies. Intervention and health-care costs were used to estimate net costs, and calculate the incremental cost per health-adjusted life-year (HALY) gained. Costs and HALYs were discounted at 3%.

**Findings:**

In the first 10 years, compliance with the WHO sodium benchmarks was estimated to avert a mean of 0·3 (95% uncertainty interval [UI] 0·2–0·5) million deaths from cardiovascular diseases and chronic kidney disease, a mean of 1·7 (95% UI 1·0–2·4) million incident cardiovascular disease events, and 0·7 (0·4–1·0) million new chronic kidney disease cases, compared with current practice. Over 10 years, the intervention was projected to be cost saving (100·0% probability), generating 1·0 (0·6 to 1·4) billion HALYs and US$0·8 (95% UI 0·3 to 1·4) million in cost savings. Over the population lifetime, the intervention could prevent 4·2 (2·4–6·0) million deaths from cardiovascular diseases and chronic kidney disease, 14·0 (8·2–20·1) million incident cardiovascular disease events, and 4·8 (2·8–6·8) new chronic kidney disease cases, with an 84·2% probability of being cost-saving and 100·0% probability of being cost-effective.

**Interpretation:**

Our modelling data suggest a high potential for compliance with WHO sodium benchmarks for packaged food being associated with substantial health gains and cost savings, making a strong case for India to mandate the implementation of the WHO sodium benchmarks, particularly as packaged food consumption continues to rise.

**Funding:**

WHO Country Office India.

## Introduction

Populations in almost all countries worldwide consume sodium above the recommended levels.^1^ This excess sodium consumption is a leading risk factor for deaths worldwide through increasing the risk of heart disease, stroke, and kidney disease.^2^ Reducing population sodium intake by 30% by 2025 is one of nine global targets recommended by WHO for the prevention and control of non-communicable diseases.^3^

Several countries including the UK, Argentina, and South Africa have shown that setting sodium content targets (or compositional limits) for packaged foods and engaging with food manufacturers to reformulate sodium levels to the targeted levels can effectively lower sodium levels across the packaged food supply, and thereby reduce population sodium intake.^4^, ^5^, ^6^ To assist more countries to implement national sodium content targets, in 2021 WHO released global sodium benchmarks for 58 packaged food groups that are major contributors to sodium intake globally.^7^ Relative to existing food industry and national sodium targets, these WHO benchmarks are substantially more comprehensive (covering more food categories) and stringent (with lower sodium content targets), and yet are feasible because they are based on existing targets from countries around the world and technical consultations.^8^ These benchmarks represent a new gold standard to aid countries to accelerate the development of their own sodium targets for packaged foods.

**Research in context**

**Evidence before this study**

High sodium consumption is the leading dietary risk of death and disability globally. WHO recommends lowering population sodium intake by reducing sodium in packaged foods, already the main source of sodium intake in high-income countries, and increasingly so in low-income and middle-income countries. In 2021, WHO released global sodium benchmarks for 58 packaged food categories to support countries in setting their own targets. At present, India has no national sodium reduction strategy, despite the population consuming double the recommended intake of sodium and increasing amounts of packaged foods. It was unclear whether mandating sodium limits for packaged foods would be a cost-effective strategy to improve health in India. We searched MEDLINE, PubMed, and Scopus databases in April, 2024, from database inception, for studies that estimated the cost-effectiveness of the WHO sodium benchmarks for packaged foods in India without language restrictions. We used the search terms: “sodium” OR “salt” AND “cost-effectiveness” AND “benchmark*” OR “target*” AND “India”. No relevant studies were identified.

**Added value of this study**

We conducted the first cost-effectiveness analysis of the mandatory implementation of the WHO sodium benchmarks for packaged foods in India using a comprehensive, multiple cohort, proportional, multistate life table (Markov) model. We used mostly India-specific data to model the change in sodium intake, health effect, health-care and implementation costs, and cost effectiveness. Our estimates suggest that mandated compliance to WHO's sodium benchmarks for packaged foods in India could avert approximately 19 million incident cases of cardiovascular and kidney disease over the population's lifetime. Such an approach has a very high likelihood of being cost saving, with a 100% probability over the first 10 and 25 years, and an 84% probability over the population's lifetime. The results were consistent across a range of sensitivity analyses altering key model assumptions and inputs, including different future trajectories of sodium intake from packaged food consumption. Notably, even if packaged food intake did not increase from baseline, mandating compliance with WHO's sodium benchmarks might still be cost saving (with >80% probability) across all the time horizons considered.

**Implications of all the available evidence**

The findings support mandating sodium limits in packaged foods in line with WHO's benchmarks as a cost-saving strategy to prevent death and disease in India, despite packaged foods not currently being the main source of sodium intake. Mandating sodium limits for packaged foods is especially important as packaged food consumption continues to rise in India.

In India, average sodium intake across the adult population is approximately double the recommended level.^9^ Unlike many high-income countries consuming a so-called western-style diet, the consumption of processed and packaged foods in India is relatively low, estimated to contribute to approximately 10% of total energy intake in 2016.^10^ Discretionary salt use (ie, salt added by the consumer when cooking or at the table) is the main source in India, making up more than 80% of sodium intake in 2014.^9^, ^11^ However, sodium intake from packaged foods is increasing as India, similar to other low-income and middle-income countries, undergoes a rapid nutrition transition.^12^ For example, the sales of salty snacks increased by 17% between 2011 and 2021,^13^ and the market for ready-to-eat products (often high in sodium) was projected to nearly triple from INR 32 billion in 2019 to INR 94 billion in 2025.^12^

Currently, there are few interventions to address high sodium intake in India, particularly sodium from increasing processed and packaged food consumption. The current national initiative, Eat Right India, launched in 2018 by the Food Safety and Standard Authority of India under the Ministry of Health and Family Welfare, aims to educate the population on reducing sodium intake as part of a broader awareness campaign about healthy eating.^14^ However, the potential effect of adopting sodium content targets for packaged foods to lower sodium intake in India is unknown. This study aimed to estimate the potential health effect, costs, and cost-effectiveness of limiting the sodium content in packaged foods in compliance with the WHO global sodium benchmarks as part of the national sodium reduction strategy in India. Understanding the potential effect, particularly in the changing nutrition landscape, can help to inform decision makers in identifying what policies represent value for money.

## Methods

### Study design, population, and data sources

We used a multiple cohort, proportional multistate, life table (Markov) model to estimate the cost effectiveness and health gains of mandating sodium content limits in packaged foods (in line with WHO's global sodium benchmarks) in India. We compared the mandatory implementation of the WHO global sodium benchmarks and the existing Eat Right India initiative to the base case scenario of the Eat Right India initiative only.^14^ The study population included all adults (all individuals aged ≥25 years) in India in 2019. The effects of the intervention were simulated over 10 years, 25 years, and over the population lifetime. The overview of the model logic pathway is outlined in the appendix (p 2), as are the data sources (appendix pp 3–4). Patient consent and ethical approval were not required because all data used were de-identified, aggregated data from existing or public databases.

### Intervention scenario

The intervention modelled was a government-led national sodium reduction strategy comprising full mandatory implementation of the WHO global sodium benchmarks for packaged foods in addition to the Eat Right India initiative.^14^ The WHO global sodium benchmarks set out maximum amounts of sodium content for different food categories.^7^ We assumed food manufacturers would gradually reduce sodium in their packaged foods and reach full compliance with the benchmarks by the end of 4 years, making 25% progress each year, based on reformulation policies in other countries.^15^ The base case and intervention scenario also accounted for the background rise in packaged food consumption in India.^12^

### Modelling change in population sodium intake

We estimated the baseline sodium intake from packaged foods in India using a food consumption survey^11^ involving 1283 individuals in north (Delhi and Haryana) and south India (Andhra Pradesh), which was done in 2014, linked with the nutritional composition data from the 2016 Indian Food Composition database^16^ and labels of packaged foods sold in Indian supermarkets; and annual sales volumes of main packaged food categories in India from 2011 to 2021 from Euromonitor (appendix pp 3–4).^17^ First, we grouped the food consumption data in the 2014 food consumption survey into food groups given by the WHO sodium benchmarks,^11^ and estimated age-specific and sex-specific total sodium intake and sodium intake from each packaged food group, adjusting for population weighting. Second, to account for the increasing sodium intake from increasing packaged food consumption over time, we predicted a food-category-specific annual percent change using sales data from 2011 to 2021 and applied it to food category-specific intakes (appendix pp 5–6).^18^

Total sodium intake was assumed to be constant over time, but the proportion coming from packaged foods was assumed to increase according to the annual percent change. In the primary analysis, we also assumed that the trend of increasing proportion of sodium coming from packaged food would continue until packaged foods accounted for 50% of the total sodium intake, and stabilise thereafter with reasons detailed in the appendix (pp 5–6).

To estimate the sodium intake of the population after the intervention, we estimated the percentage reduction in the sodium content of packaged foods within a category after reformulation to meet the WHO benchmark levels, compared with the base case scenario (ie, no reformulation), following previously established methods.^8^ Using data collected in 2018 on brand-specific and product-specific packaged foods in India,^19^ three researchers (KT, LH, and SRT) mapped the brand-specific and product-specific sodium data in India to the WHO global sodium benchmark food categories. Under full implementation of the WHO benchmarks, it was assumed that all foods exceeding the benchmarks were reformulated to the benchmark sodium level within 4 years. We then calculated the proportional reduction of each individual product and estimated the average proportional reduction for each food category targeted by WHO benchmarks. The proportional reduction in sodium content after reformulation for each food category was then applied to the age-specific and sex-specific estimates of the corresponding food groups from the dietary survey to estimate the reduction in sodium intake from each food category. The total reduction in sodium intake after intervention was calculated as the sum of the sodium reduction from all WHO-targeted food groups in the dietary survey data, accounting for the increasing proportion of sodium consumption from packaged food over time.

### Modelling health outcomes

We used age-specific and sex-specific estimates of incidence, prevalence, and mortality rates of cardiovascular diseases, including ischaemic heart disease, ischaemic stroke, intracerebral and subarachnoid haemorrhage, atrial fibrillation and flutter, and hypertensive heart disease, as well as chronic kidney disease for the 2019 Indian population from the Global Burden of Diseases, Injuries, and Risk Factors (GBD) Study 2019 study to populate our model (appendix pp 3–4).^20^, ^21^

We used DisMod II software^22^ to enforce internal consistency in the chronic kidney disease and cardiovascular disease epidemiological estimates obtained from the GBD 2019 study, and derived case fatality rates that were not provided in the GBD data.^22^ We used age-specific and sex-specific estimates of mean systolic blood pressure from the GBD study and distributions from an India-specific study.^23^, ^24^ The blood pressure effect of reduced sodium intake was derived from a meta-analysis of 85 sodium reduction trials with a duration of at least 4 weeks.^25^ Age-specific relative risks of chronic kidney disease and cardiovascular disease outcomes due to systolic blood pressure were derived from a meta-analysis of large cohort studies undertaken for the 2019 GBD study.^21^, ^26^ We calculated disability weights using disease-specific estimates of prevalence and years lived with disability from the 2019 GBD study.^20^, ^27^

### Intervention and health-care costs

An extended health-care perspective was used, incorporating direct health-care costs of modelled diseases, costs for all other health care, and costs for policy implementation. Resource needs and India-specific intervention costs associated with mandating and monitoring the WHO global sodium benchmarks as part of a sodium reduction strategy were derived from the WHO Non-communicable Disease Costing Tool.^28^ We included costs for the first 4 years of implementation, which involved costs of human resources (related to legislation and monitoring compliance), training workshops, meetings, mass media campaigns (to raise awareness of the importance of sodium reduction and generate acceptability for the policy), and supplies and equipment. An ongoing cost for annual law enforcement and inspection (monitoring sodium amounts in packaged foods) was also included in the model after 4 years.

We conducted a literature search to identify the best estimates of the total health expenditure and health-care costs for chronic kidney disease and major cardiovascular diseases in India (appendix pp 3–4). The age-specific and sex-specific costs of all other diseases were calculated as the difference between total health-care expenditure and the chronic kidney disease-specific and cardiovascular disease-specific health-care costs for each age and sex group. Details of the calculation of costs are shown in the appendix (pp 3–4). All costs were estimated in the local currency, then inflated to 2019 values using the World Bank consumer price index for India.

### Cost-effectiveness modelling

We used The Population Health and Cost-Effectiveness Simulation Model (PHACES), a multiple cohort proportional multistate life table (Markov) model, to estimate the incremental costs and health outcomes over 10 years, 25 years, and the population lifetime, by comparing the base case scenario with the intervention scenario. Details of PHACES, the modelling approach, and input data are provided in the appendix (pp 7–10).

To calculate the incremental cost-effectiveness ratio (ICER), we divided the incremental net cost (ie, the sum of policy and health-care costs) by the incremental health-adjusted life-years (HALYs) gained. Both costs and HALYs were discounted at 3% annually. All estimated costs were quantified in 2019 Indian rupees and US dollars, with an exchange rate of INR 68·97 per US$ (as per July 1, 2019), as reported by the International Monetary Fund.^29^ Cost saving was defined as having a negative incremental net cost, and a recently estimated India-specific threshold based on life expectancy and health expenditure (ie, $487 per HALY gained) was used to define a cost-effective intervention.^30^

The variable uncertainty around the modelled estimates was quantified using Monte Carlo simulations (n=1000). For each iteration, a draw was made from the distributions of mean reduction of sodium intake, the dose–response relationship between sodium reduction and blood pressure change, relative risks, health-care costs, and policy implementation costs. The point estimate and 95% uncertainty intervals (UIs) were defined as the mean and 2·5th to 97·5th percentiles, respectively. The cost-effectiveness analysis was conducted using RStudio version 2023.06.0.

### Sensitivity analyses

We assessed the following alternative assumptions: (1) that it took a 10-year implementation period (rather than 4 years) to reach the WHO global sodium benchmarks, in which both the gradual reduction of sodium intake and policy implementation costs were assumed to span across the initial 10 years of the policy; (2) that the trend in increasing sodium from packaged foods continued until sodium intake from packaged foods contributed to 80% (compared with 50% in the primary model) of total sodium intake as seen in high-income countries; (3) that there was a slower increase in packaged food consumption based on fitting Euromonitor per person sales volumes to a linear trend, rather than using the annual percentage change; (4) that there was no further increase in packaged food consumption beyond that observed in 2019; (5) that there was a 0% discount rate of health effect and costs; and (6) that there was a 5% discount rate of health effect and costs.

### Role of the funding source

The funder of the study had no role in study design, data collection, data analysis, data interpretation, or writing of the report.

## Results

If full compliance with the WHO sodium benchmarks were reached in India, our model showed that this could lower mean sodium intake by 138 mg per day in women and 184 mg per day in men after 4 years of implementation (table 1). This corresponded to a 21% and 19% reduction in pre-intervention sodium intake levels from packaged foods for women and men, respectively; or a 5% and 6% reduction in total sodium intake, respectively.

**Table 1:** Estimated sodium intake from packaged foods before reformulation and reductions in sodium intake after reformulation to WHO sodium benchmarks by sex and year

	Sodium intake from packaged foods in the base case scenario (ie, before reformulation)*		**Sodium intake from packaged foods in the policy scenario (ie, after reformulation)**†	
	Mean intake (mg per day)	Proportion (%) of total sodium intake	Mean intake (mg per day)	Reduction compared with base case scenario (mg per day)
**Women**				
Year 0 (baseline)	479	17%	479	0
Year 1	518	18%	492	26
Year 2	561	20%	505	56
Year 3	609	22%	516	93
Year 4	664	24%	526	138
**Men**				
Year 0 (baseline)	689	20%	689	0
Year 1	747	22%	713	34
Year 2	812	24%	737	75
Year 3	884	26%	760	124
Year 4	965	28%	781	184

* Sodium intakes before reformulation at baseline and the 4 subsequent years were derived from a dietary survey, an Indian food composition database, and annual sales data of packaged foods to account for the increasing sodium intake from increasing packaged food intake over time.

† Sodium intakes after reformulation were estimated by applying the percentage reduction in the sodium content of packaged foods after reformulation to the WHO benchmark levels, to sodium intakes before reformulation. Sodium content before reformulation was derived from brand and product-specific packaged food data in India in 2018. The SDs of the mean sodium intake at baseline (in 2019) could not be estimated because they were generated on the basis of summary data on dietary intake in 2014. Although 2014 sodium intakes were estimated on the basis of individual-level dietary survey data, we used summary data to inflate the mean intake to 2019 using food category-specific annual percentage changes generated from annual food sales data from 2011 to 2021.

After full compliance with the WHO benchmarks after 4 years, the absolute reductions in sodium intake were estimated to continue to grow (appendix p 11), given the assumed increasing trend of packaged food consumption and contribution to total sodium intake (appendix pp 12–13). Of individual packaged food categories, the largest sodium intake reductions after reformulation were estimated for comminuted meat products; processed fish products (eg, dried salted fish); raw meat products and preparations; and snacks (appendix p 13). In 43 of the 49 packaged food categories affected by the WHO sodium benchmarks consumed by the Indian population, most products (≥50%) exceeded the benchmarks (appendix pp 14–16).

Mandatory compliance to WHO's sodium benchmarks for packaged foods in India was estimated to avert a mean of 1·7 (95% UI 1·0–2·4) million incident cardiovascular disease events and 0·7 (0·4–1·0) million new chronic kidney disease cases in the first 10 years, a mean of 7·6 (4·4–11·0) million and 2·7 (1·6–3·9) million, respectively, after 25 years, and a mean of 14·0 (8·2–20·1) million and 4·8 (2·8–6·8) million, respectively, over the population lifetime (table 2; appendix p 17). In the same time periods, the intervention was estimated to avert a substantial number of cardiovascular disease and chronic kidney disease deaths: a mean of 0·3 (95% UI 0·2–0·5) million after 10 years, 2·4 (1·4–3·5) million after 25 years, and 4·2 (2·4–6·0) million over the population lifetime (table 2; appendix p 18). Although most averted cardiovascular disease events were due to a reduced ischaemic heart disease burden, a considerable portion of the averted deaths were due to stroke (appendix pp 18–19). The number of HALYs gained were estimated to be a mean of 1·0 (95% UI 0·6–1·4) million after 10 years, 11·1 (6·4–16·0) million over 25 years, and 35·9 (20·9–51·3) million over the population lifetime (table 2; appendix p 19). 42·2% (95% UI 41·9–42·5) of the estimated health gains accrued to women over the population lifetime (appendix p 19). The observed lower estimated health gains accrued among women compared with men is likely due to lower sodium intake from packaged foods and lower cardiovascular disease burden in women compared with men.

**Table 2:** Estimated health gains, costs, and incremental cost-effectiveness ratio of implementing the WHO sodium benchmarks for packaged foods in India

		**10 years**	**25 years**	**Population lifetime**
Incident cardiovascular disease events averted, millions		1·7 (1·0 to 2·4)	7·6 (4·4 to 11·0)	14·0 (8·2 to 20·1)
Incident chronic kidney disease cases averted, millions		0·7 (0·4 to 1·0)	2·7 (1·6 to 3·9)	4·8 (2·8 to 6·8)
Averted cardiovascular disease and chronic kidney disease deaths, millions		0·3 (0·2 to 0·5)	2·4 (1·4 to 3·5)	4·2 (2·4 to 6·0)
Health-adjusted life years gained, millions		1·0 (0·6 to 1·4)	11·1 (6·4 to 16·0)	35·9 (20·9 to 51·3)
Health-care costs, billion US$				
	Modelled diseases (ie, cardiovascular disease and chronic kidney disease)	−1·0 (−1·7 to −0·5)	−5·2 (−8·7 to −2·6)	−10·5 (−17·1 to −5·7)
	Other health care	0·1 (0·1 to 0·2)	1·8 (0·9 to 3·0)	7·9 (3·7 to 13·5)
	Total	−0·9 (−1·5 to −0·4)	−3·4 (−6·6 to −1·3)	−2·8 (−9·1 to 1·9)
Policy implementation costs, billion US$		0·1 (0·1 to 0·1)	0·1 (0·1 to 0·2)	0·1 (0·1 to 0·2)
Net costs, billion US$		−0·8 (−1·4 to −0·3)	−3·3 (−6·4 to −1·2)	−2·5 (−8·2 to 2·7)
ICER, US$ per HALY gained		Dominant* (dominant to dominant)	Dominant* (dominant to dominant)	Dominant* (dominant to 71·4)
Probabilities				
	Cost saving†	100·0%	100·0%	84·2%
	Cost effective‡	100·0%	100·0%	100·0%

Data are mean (95% uncertainty interval). Chronic kidney disease and cardiovascular disease included ischaemic heart disease, ischaemic stroke, intracerebral and subarachnoid haemorrhage, atrial fibrillation and flutter, and hypertensive heart disease. HALY=health-adjusted life-year. ICER=incremental cost-effectiveness ratio.

* An estimate reported as dominant indicates the intervention was both more effective and less costly compared with the base case scenario. Thus, estimates with positive health gains and negative net costs are reported as dominant.

† Cost saving was defined as having a negative incremental net cost.

‡ Cost effective was defined as US$487 per HALY gained based on Pichon-Riviere and colleagues.^30^

**Figure 1:**
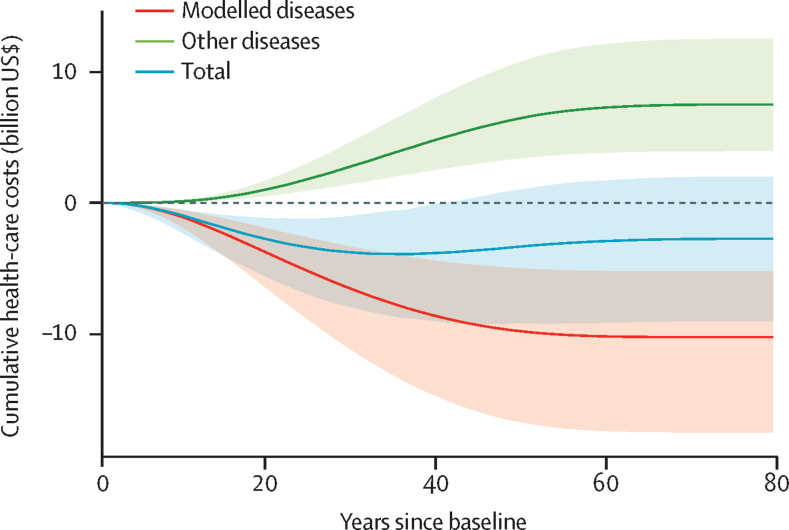
Annually updated cumulative estimates of health-care costs if the WHO global sodium benchmarks for packaged foods were implemented in India The modelled diseases were cardiovascular disease and chronic kidney disease. Increased costs were estimated for other diseases (ie, not cardiovascular disease and chronic kidney disease) due to increased survival because the intervention would reduce cardiovascular disease and chronic kidney disease mortality. Lines and shaded areas represent means and 95% uncertainty intervals, respectively, of 1000 Monte Carlo simulations.

The intervention was estimated to generate $1·0 (95% UI 0·5–1·7) billion (INR 69 billion) in health-care cost savings related to chronic kidney disease and cardiovascular diseases after 10 years, $5·2 (2·6–8·7) billion (INR 360 billion) after 25 years, and $10·5 (5·7–17·1) billion (INR 726 billion) over the population lifetime (table 2; figure 1). The increased survival from the reduced mortality from cardiovascular diseases and chronic kidney disease was estimated to result in increased costs for other health care: a mean of $0·1 (95% UI 0·1–0·2) billion (INR 6·9 billion) in the first 10 years, $1·8 (0·9–3·0) billion (INR 124·1 billion) after 25 years, and $7·9 (3·7–13·5) billion (INR 544·9 billion) over the population lifetime.

In the first 10 years, the intervention costs were estimated to amount to a mean of $0·10 (95% UI 0·07–0·14) billion (INR 7 billion), increasing to $0·12 (0·09–0·16) billion (INR 8 billion) after 25 years, and $0·14 (0·11–0·18) billion (INR 10 billion) across the population lifetime (table 2). The most expensive component of the intervention was the mass media needed to educate the population about the need to reduce sodium intake and increase public acceptability for the mandatory policy (appendix p 20).

**Figure 2:**
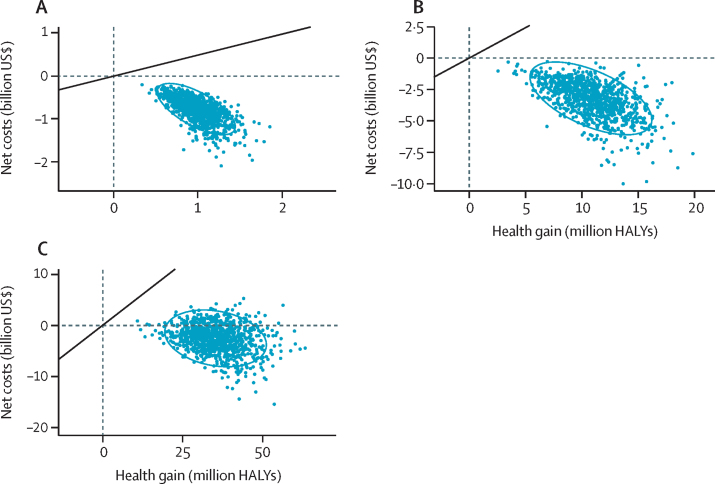
Cost-effectiveness plane with net costs (ie, health-care costs plus policy costs) plotted against HALYs gained after 10 years, 25 years, and over the population lifetime (A) 10 years. (B) 25 years. (C) Population lifetime. The blue dots represent model estimates from 1000 Monte Carlo simulations and the blue ellipses represent the two-dimensional 95% uncertainty region centred around the means. Estimates with negative costs indicate a cost-saving intervention. The solid diagonal line represents the thresholds for a cost-effective intervention (ie, US$487 per HALY gained^30^). Both HALYs and net costs were discounted by 3% per year. HALY=health-adjusted life-year.

Over the first 10 and 25 years after implementation, the policy had a 100·0% probability of being cost saving, generating greater total health-care savings than implementation costs (table 2; figure 2). The policy was also likely (84·2% probability) to be cost saving over the population lifetime. Regardless of the time horizon, the policy had a 100·0% probability of being cost effective (table 2; figure 2).

**Figure 3:**
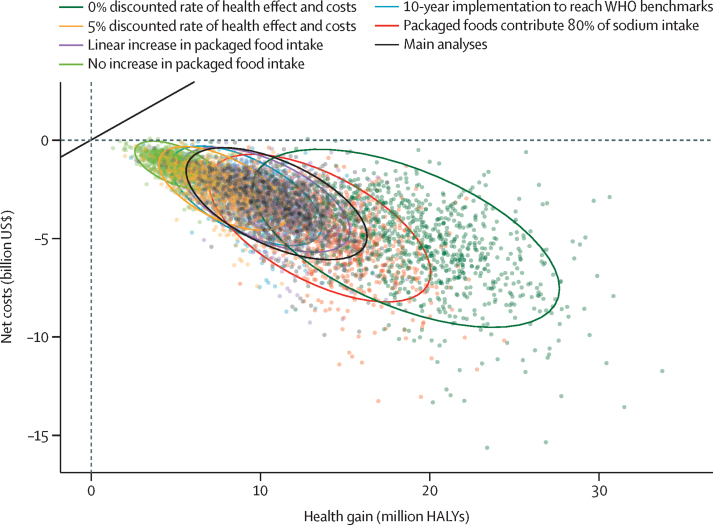
Cost-effectiveness plane with net costs (ie, health-care costs plus policy costs) after 25 years plotted against HALYs gained in the same time period, estimated in the main analysis and deterministic sensitivity analyses The dots represent model estimates from 1000 Monte Carlo simulations and the ellipses of the same colour represent the two-dimensional 95% CIs centred around the mean. Estimates with negative costs indicate a cost-saving intervention. The solid diagonal lines represent the threshold for a cost-effective intervention (ie, $487 per HALY gained). HALY=health-adjusted life-year.

The magnitude of net costs and health gains differed between the different sensitivity analyses, but both ICER and probabilities of the intervention being cost saving or cost effective were similar to the main analysis (figure 3; appendix p 21). In all sensitivity analyses, the probability of the intervention being cost saving in the first 25 years was 99·8% or higher (figure 3; appendix p 21). Assumptions regarding future packaged food consumption amounts (ie, 80% contribution to total sodium intake, or slower increase in packaged food consumption over time, or no increase in packaged food consumption) had a considerable effect on estimated health gains over the population lifetime, but the ICER and cost saving and cost effectiveness probabilities were similar to the main analysis (appendix p 21). As expected, assumptions regarding discount rate had the greatest effect on ICER and the probabilities of cost saving over the population lifetime (appendix p 21).

## Discussion

Using Markov cohort models with nationally representative data, we estimated that the mandatory implementation of the WHO global sodium benchmarks for packaged foods in India is likely to be a cost-saving intervention to prevent cardiovascular diseases and chronic kidney disease, across all the timeframes considered. The findings were consistent across a series of sensitivity analyses testing the effect of differing underlying model assumptions, including future trajectories in packaged food consumption in India.

To the best of our knowledge, our findings are novel, with most previous modelling studies on sodium reformulation policies being conducted in high-income countries, where the absolute and proportional intake of sodium from packaged foods is already higher relative to India.^8^, ^31^ For instance, in Australia and the USA, where sodium reformulation policies have been estimated to have a high likelihood of being cost saving, the population consumes approximately 2500 mg per day of sodium from packaged foods, which is roughly three times higher than India.^8^, ^11^, ^31^, ^32^ Our findings add to these previous observations and provide strong support for the implementation of the WHO global sodium benchmarks for packaged foods in India as an immediate measure to counter the escalating sodium intake from these packaged foods. Notably, our evaluation suggested that even if the intake of packaged foods in India did not change, the mandatory implementation of the WHO sodium benchmarks for packaged foods was still likely to be cost saving. Although based on observed trends in packaged food consumption, we consider such a scenario to be highly unlikely.

Implementing mandatory sodium limits for packaged foods in India at present, when the consumption of packaged foods is relatively low, is likely to be more feasible than when packaged foods form a large portion the population's diet, as seen in many higher-income countries.^6^, ^33^ The difficulty in implementing such limits once packaged food consumption increases is due to the probability of there being more food companies that need to be engaged in sodium reformulation and additional packaged food categories for which sodium content targets would need to be set. South Africa and Argentina were the first among nine countries to implement mandatory sodium targets for a range of packaged foods, suggesting the feasibility of mandatory sodium limits.^6^ Additionally, the benefit of implementing sodium limits for packaged foods at present is that as more packaged foods are produced or imported into India over time, food companies will have an established understanding of the maximum sodium amount in foods to comply with, and the lower sodium amounts will be the market norm. To improve the feasibility of implementing the WHO global sodium benchmarks, it is important to adapt it to the local Indian diet and target Indian-specific foods. In recognition of the need for local adaption, WHO have since published the southeast Asia region sodium benchmarks and a second edition of the global benchmarks that incorporate the southeast Asia region benchmarks.^34^, ^35^ The second edition of the WHO global sodium benchmarks includes new category benchmarks and mostly lower (stricter) sodium targets; thus our findings, which are based on the original benchmarks, are conservative.

Our analysis has various strengths. First, most of the data used were contemporary and specific to India, including data on dietary intake, packaged food content and sales volumes, intervention costs, blood pressure, cardiovascular disease and chronic kidney disease rates, and most health-care cost data. Second, we used longitudinal food sales data to account for the increasing trend in packaged food consumption to better capture the changing dietary patterns in India. Third, the relationship between sodium and blood pressure was based on the most recent meta-analysis of sodium reduction trials. Fourth, we used relative risk estimates from meta-analyses of large observational studies. Fifth, where possible, we used sex-specific data, which allowed us to estimate health effects for women and men separately. Sixth, we included health-care expenditure for modelled diseases (cardiovascular disease and chronic kidney disease) and other health-care expenditures. Finally, we conducted several sensitivity analyses using different assumptions to assess the robustness of our findings.

There are also several limitations of our study that should be considered. First, the dietary intake data were collected from Andhra Pradesh, Delhi, and Haryana and are not nationally representative. However, there were various strengths that justify their use, including the large sample size; inclusion of urban slum, urban, and rural populations in north and south of India; application of population weighting to reflect the population structures of the areas surveyed; and use of rigorous dietary assessment methods (two 24-h diet recalls using the gold standard five-pass method). Second, the dietary intake data were from 2014, and there were too few data to accurately estimate future food consumption patterns in India given the ongoing nutrition transition towards increased packaged food consumption. For example, it is unclear at what point the increase in packaged food consumption will plateau. However, we used conservative estimates in our primary analysis and used amounts observed in high-income countries (ie, 80% of total sodium intake from packaged foods) in our sensitivity analyses. Our predictions of future trends in packaged food consumption were based on annual per person sales estimates from 2011 to 2021, which might not fully capture actual food intake and are limited by the small number of data points (ie, one estimate per year and food category over 11 years). Despite this, we estimated food category-specific trends; visually inspected, predicted, and observed sales data to ensure no violations of assumptions in trend prediction; and conducted multiple sensitivity analyses to confirm the robustness of our findings. Third, because of the absence of empirical evidence about the effect of mandating sodium benchmarks in packaged foods in India, we assumed no change in population choices of foods after reformulation. Sodium reformulation experiences in other countries suggest food manufacturers can make gradual reductions in sodium content in packaged foods without consumers noticing.^4^ Fourth, although most data on health-care costs came from Indian sources, annual costs for atrial fibrillation care were derived from Brazil as the next best available data. Deviations in atrial fibrillation costs will minimally affect the findings given that health gains related to atrial fibrillation only represented a small proportion of the total health gains. Fifth, we did not include indirect costs, such as productivity loss, because of absenteeism and disability or productivity gains associated with averted deaths and new cases of cardiovascular disease and chronic kidney disease, meaning that the potential cost savings from a societal perspective are probably underestimated. Sixth, we did not incorporate the population trends on incidence and mortality of cardiovascular disease and chronic kidney disease in the model, given the absence of any clear age-standardised trends downwards or upwards. Seventh, our estimates of the health benefits of reducing sodium intake are conservative, and do not include other potential health outcomes that are not mediated by blood pressure (for example stomach cancer); however, such health outcomes are likely to be few.^36^ Eighth, there might be model misspecification bias because of the absence of access to individual level data for some inputs (eg, relative risks of cardiovascular disease and chronic kidney disease) and relying on parametric bootstrapping. Finally, our study does not prove the cost effectiveness of complying with WHO global sodium benchmarks for packaged foods, but rather it provides decision makers with quantitative estimates, and corresponding uncertainty, of the potential effect and cost effectiveness that can inform the development of sodium reduction policies in India and other low-income and middle-income countries.

Compared with a base scenario without any restrictions to sodium in packaged foods, implementing and complying with WHO sodium benchmarks for packaged foods could help prevent millions of deaths and incident cardiovascular disease and chronic kidney disease events over the population lifetime, and has high potential to be cost saving in India. Our findings support the mandatory implementation of the WHO sodium benchmarks for packaged foods in India, especially as packaged food consumption is rising.

### Contributors

### Data sharing

All data relevant to the study are included in the Article. Original data are available upon reasonable request. Requests for data should be directed to Kathy Trieu (ktrieu@georgeinstitute.org.au) and will be reviewed by the research team.

## Declaration of interests

KT is co-Director of the WHO Collaborating Centre on Salt Reduction. LH reports grant support from the Australian National Health and Medical Research Council, outside of the present study. LNA is supported by an Australian National Health and Medical Research Council Investigator Grant and an Honorary Heart Foundation Fellowship. MM reports grant support from Resolve To Save Lives and Northwestern University, and travel support from the Nordic Dairy Congress 2022, all outside of the present work. RG is a staff member of WHO, and the authors alone are responsible for the views expressed in this publication, and they do not necessarily represent the views, decisions, or policies of WHO. All other authors declare no competing interests.
