# Supplementary material for: Estimated health benefits, costs, and cost-effectiveness of implementing WHO's sodium benchmarks for packaged foods in India: a modelling study
Source: Lancet Public Health. 2024 Oct 30;9(11):e852–60. doi: 10.1016/S2468-2667(24)00221-4 (PMC11535755; doi:10.1016/S2468-2667(24)00221-4)
Supplement: Supplementary appendix [file mmc1.pdf]

THE LANCET
Public Health

Supplementary appendix

This appendix formed part of the original submission and has been peer reviewed. We post it as supplied by the authors.

Supplement to: Trieu K, Huang L, Aminde LN, et al. Estimated health benefits, costs, and cost-effectiveness of implementing WHO's sodium benchmarks for packaged foods in India: a modelling study. *Lancet Public Health* 2024; **9:** e852–60.

Estimated health benefits, costs, and cost-effectiveness of implementing WHO's sodium benchmarks for packaged foods in India: a modelling study – Supplementary Appendix

Kathy Trieu, Liping Huang, Leopold N Aminde, Linda Cobiac, Daisy H Coyle, Mary Njeri Wanjau, Sudhir Raj Thout, Bruce Neal, Jason HY Wu, Lennert Veerman, Matti Marklund* Rachita Gupta*

Table of Contents

Supplementary File 1. Overview of the logic pathway for modelling the incremental cost-effectiveness ratio of compliance to WHO's global sodium benchmarks for packaged foods........... 2

Supplementary File 2. Details of input data sources used to populate the model.............................. 3

Supplementary File 3. Estimating future trends in packaged food consumption............................... 5

Supplementary File 4. Population Health and Cost-Effectiveness Simulation Model (PHACES)..... 7

Supplementary File 5. Sodium intake, systolic blood pressure and relative risks input data 9

Supplementary File 6. Impact of reformulation in compliance with WHO global benchmarks on sodium intake by sex over 30 years 11

Supplementary File 7. Annual percentage change (%) of consumption for different packaged food groups from 2011 to 2021.. 12

Supplementary File 8. Top ten largest sodium reduction (mg/day) post-reformulation at Year 4 by WHO global sodium benchmark food categories.. 13

Supplementary File 9. Mean sodium levels in packaged foods in India compared against WHO global sodium benchmarks 14

Supplementary file 10. Annually updated cumulative estimates of averted new cases of total cardiovascular diseases 17

Supplementary File 11. Annually updated cumulative estimates of averted deaths from total cardiovascular diseases (A), ischemic heart disease (B), stroke (C), atrial fibrillation (D), hypertensive heart disease (E), and chronic kidney disease (F), if the WHO global sodium benchmarks for packaged foods were achieved in India. 18

Supplementary File 12. Annually updated cumulative estimates of health-adjusted life years (HALYs) gained in A) the total adult Indian population, and B) separately in women (red) and men (blue), if the WHO global sodium benchmarks for packaged foods were achieved in India. 19

Supplementary File 13. Government intervention costs of legislating and monitoring the WHO global sodium benchmarks in India based on 2019 costs (Indian rupees)....................................... 20

Supplementary File 14. Health-adjusted life years (HALYs) gained, net costs, and incremental cost-effectiveness ratios estimated over the first 10 years, 25 years and population lifetime in the main analysis and deterministic sensitivity analyses 21

Supplementary File 1. Overview of the logic pathway for modelling the incremental cost-effectiveness ratio of compliance to WHO's global sodium benchmarks for packaged foods. CKD, chronic kidney disease; CVD, cardiovascular disease; HALY, Health-adjusted life year; ICER, incremental cost-effectiveness ratio.

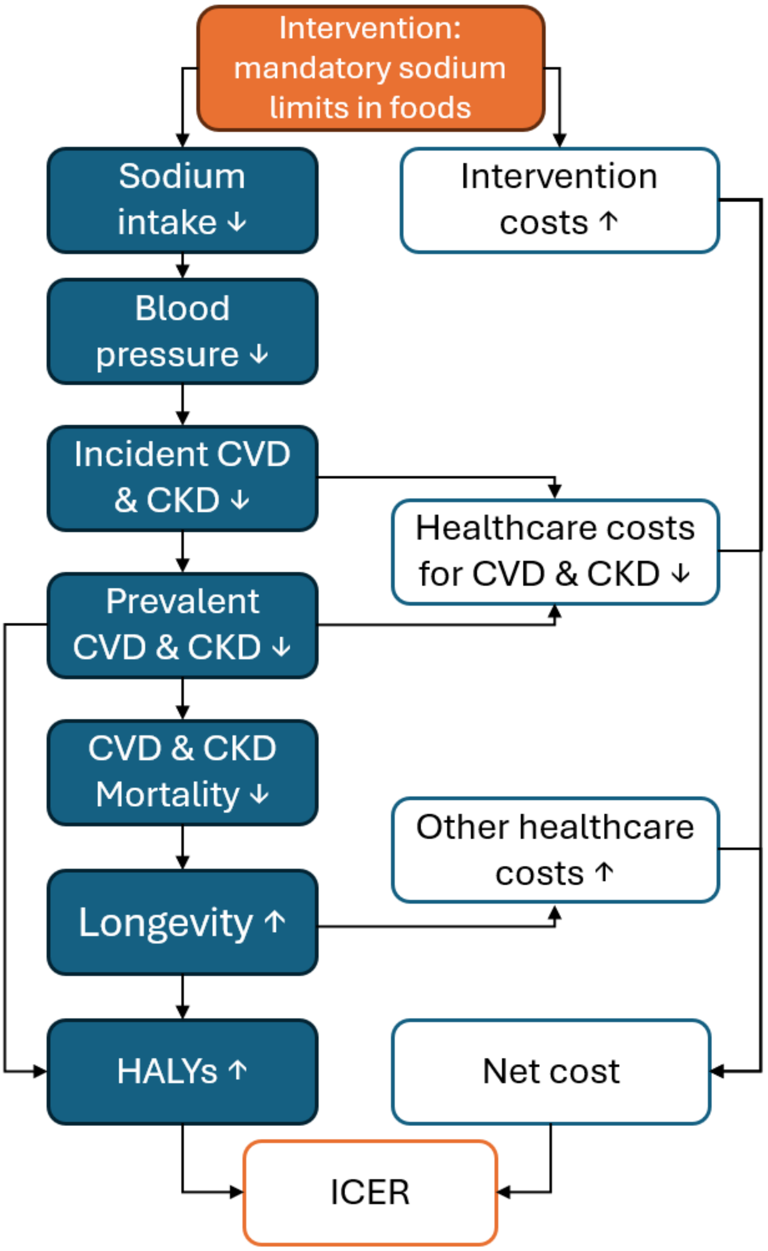

Supplementary File 2. Details of input data sources used to populate the model

Data	Categories	Details of data, its use and source
Food intake (g/d) and sodium intake from different foods (mg/d)	5-year age and sex groups	Two five-pass 24-hour dietary recalls conducted in 2014 from 1,283 participants aged 20 years and older living in rural, urban and slum areas of Andhra Pradesh, Delhi, and Haryana (Johnson et al[1]) with weighting to reflect the population structure of the respective regions.<br>Nutritional composition of foods (sodium content) were based on the 2016 Indian food composition tables and supplementary data from labels of packaged foods sold in Indian supermarkets[2].<br>We used 24-hour dietary recall and nutrition compositional data to estimate pre-reformulation sodium intake in total and for each packaged food category for each age-sex group in 2014.
Packaged food sales volume and population size in India from Euromonitor	WHO packaged food groups	Euromonitor's annual estimates of processed and packaged food sales volume and Indian population size from 2011 to 2021 (Euromonitor[3]).<br>The observed increasing trend in packaged food sales volumes per capita was used to adjust 2014 estimates of sodium intake from packaged foods to baseline and onwards pre-reformulation.
Sodium content of brand-specific foods (mg/100g)	WHO packaged food groups	Sodium content in packaged foods targeted by the WHO global sodium benchmarks were obtained from nutrition labels of 2,725 packaged foods out of ~15,000 product-specific packaged foods collected in India in 2018 (India FoodSwitch[4]).<br>We used the sodium content data to calculate pre- and post- reformulation sodium content of packaged foods targeted by the World Health Organization (WHO) sodium benchmarks and estimated the average proportional reduction of each targeted food group.
Blood pressure data	5-year age and sex groups	We used age-and-sex specific estimates of mean systolic blood pressure (SBP) from the Global Burden of Disease (GBD) study,[5,6] and assumed the standard deviation of SBP in each age-sex-stratum would range 12-17% of the mean, depending on the strata, based on age- and sex-specific systolic blood pressure (SBP) distributions measured in over 180,000 Indian adults.[7]
Relative risks	5-year age and sex groups	Relative risks for cardiovascular disease (CVD) and chronic kidney disease (CKD) for change in SBP from GBD 2019 study and meta-analysis of cohort studies[5,8,9]
Sodium-BP relationship	All ages, by hypertension status	Meta-analysis of experimental studies which estimated a mean reduction in systolic blood pressure of 5.56 mmHg (95% CI:4.52; 6.59) per 100 mmol/day reduction in sodium intake.[10]
CVD and CKD epidemiology	5-year age and sex groups	Data on incidence, prevalence and mortality rates were based on India-specific estimates from the GBD 2019 study.[5,6] The GBD study utilizes various primary data sources for each country. The list of primary data sources is publicly available. These were entered in the DisMoD II software to derive case fatality rates.[11] We calculated disability weights using disease-specific estimates of prevalence and years lived with disability from the 2019 GBD study.
Population numbers and overall mortality rates	5-year age and sex groups	Population numbers and overall mortality rates in the baseline year were from GBD 2019 study[5,6]
CVD and CKD specific healthcare costs	5-year age and sex groups	Annual costs for CKD patients not requiring dialysis were derived from a study conducted within the STOP CKDu (Study to Test and Operationalize Preventive approaches for CKD of undetermined aetiology) research program in Uddanam.[12] Annual costs of CKD among dialysis patients were based on claims data for patients receiving hemodialysis care under The Rajiv Aarogyasri Community Health Insurance Scheme (RACHIS).[13] To derive annual average costs for all CKD patients, we calculated the mean of costs for CKD patients without dialysis and patients on dialysis, weighted for the prevalence of dialysis among CKD patients in India (i.e., 0.04%).[14] Costs per incident IHD case and annual costs per prevalent IHD case were based on

		estimates from WHO-CHOICE.[15] Costs per incident stroke and annual costs per prevalent stroke were informed by data from the 2017-18 National Sample Survey Organization survey.[16] The annual costs for prevalent atrial fibrillation were based on a community-based Brazilian cohort study,[17] while accounting for between-country differences in total healthcare expenditure per capita. Annual costs for prevalent hypertensive heart disease were derived from a study conducted in a hypertension clinic of Nehru Hospital, a tertiary care centre (Postgraduate Institute of Medical Education and Research (PGMER)) in Chandigarh.[18]<br>The CKD- and CVD-specific healthcare costs were calculated using the costs of incident and/or prevalent cases, the CKD and CVD incidence and prevalence rates, and population size of the age-sex group.
Total health expenditure	5-year age and sex groups	Data on Total Health Expenditure (THE) was obtained from the India National Health Accounts 2018-19, and distributed across age and sex groups using India-specific age patterns for health expenditure reported in *Implications of Age profile of healthcare expenditure and age structural transition on future healthcare spending in India.*[19,20]<br>Sex-specific THE estimates were distributed across age groups using THE per capita age pattern in India.
Intervention costs	-	Costs to government for the implementation of the policy were derived from the WHO NCD Costing Tool[21] (see Supplementary file 12)

Supplementary File 3. Estimating future trends in packaged food consumption

Food category-specific annual percent changes (APC) were estimated from per capita packaged food sales volumes of 21 individual food categories. These were derived from packaged foods sales volume and Indian population size data between 2011 and 2021 reported by Euromonitor.[3] For each food category, we estimated the APC by fitting a simple linear model. First, we regressed the natural logarithm of the yearly per capita sales volume (y_c) of category c on time t, according to equation 1.

$$E(\ln(y_c)) = \alpha_c + \beta_c t \qquad \text{Eq 1.}$$

The APC was then calculated according to Equation 2, where $\hat{\beta}$ is the estimated slope obtained from the regression model in equation 1.

$$APC_c(\%) = 100\left(e^{\hat{\beta}_c} - 1\right) \qquad \text{Eq 2.}$$

Total sodium intake was assumed to be constant although sodium from packaged foods would increase according to the APC. That is, we assumed that the sodium contribution from packaged foods would increase together with a proportional reduction in sodium intake from discretionary salt, representing a temporal shift in sources of dietary sodium. In the primary analysis, we assumed that the trend in the shift from discretionary salt to packaged foods would continue until packaged foods account for 50% of total sodium intake, and stabilize thereafter. This is because other foods, such as those prepared and consumed within the home and outside of the home (e.g. by the informal food sector or restaurants), are significant contributors to sodium intake in India and are unlikely to be completely replaced by packaged foods.[22,23] This is the case in several high-income countries such as Japan, Singapore, Brunei and Portugal whereby packaged foods contribute between 40% and 60% of total sodium intake.[24]

We also considered an alternative approach, the average annual percentage charge (AAPC), to estimate the future trend in packaged food consumption, as it has advantages if the data cannot be fitted to a linear model. The AAPC is a summary measure to describe the weighted average trend over an interval that can be broken down into multiple segments, each with its own APC, to better fit data.[25] We used the Joinpoint Regression Program (Version 5.0.2 - May 2023; Statistical Methodology and Applications Branch, Surveillance Research Program, National Cancer Institute) to simultaneously calculate the conventional APC (cAPC) described in Equations 1-2 and the AAPC, under various assumptions regarding the correlation of random errors in the regression model (uncorrelated, first order autocorrelation estimated from the data, or autocorrelation with correlation=0.2). However, given the largely monotonic trends in the Euromonitor packaged food sales data over the period 2011-2021, the two methods (cAPC and AAPC) provided very similar estimates of annual change, with little impact of the assumptions regarding autocorrelation. Thus, we used the simpler APC approach described above to predict future intakes of packaged foods.

We conducted an assessment of the assumptions underlying our regression model by examining residual plots to evaluate linearity and homoscedasticity. While the small sample size (11 data points per food category) limits the power of these tests, we believe that our checks reasonably assure that the assumptions of normality and homogeneity of variance were not grossly violated. To further ensure the robustness of our findings, we explored the impact of alternative assumptions regarding the correlation of random errors in the regression model, including uncorrelated errors, first-order autocorrelation, and fixed autocorrelation (correlation coefficient = 0.2). The estimated APC remained consistent across these scenarios, with differences of less than 10%. This consistency, coupled with sensitivity analyses, demonstrates that our conclusions—that the intervention is cost-effective over the long term and potentially cost-saving within the first 10 years—are robust, even if some assumptions were not fully met.

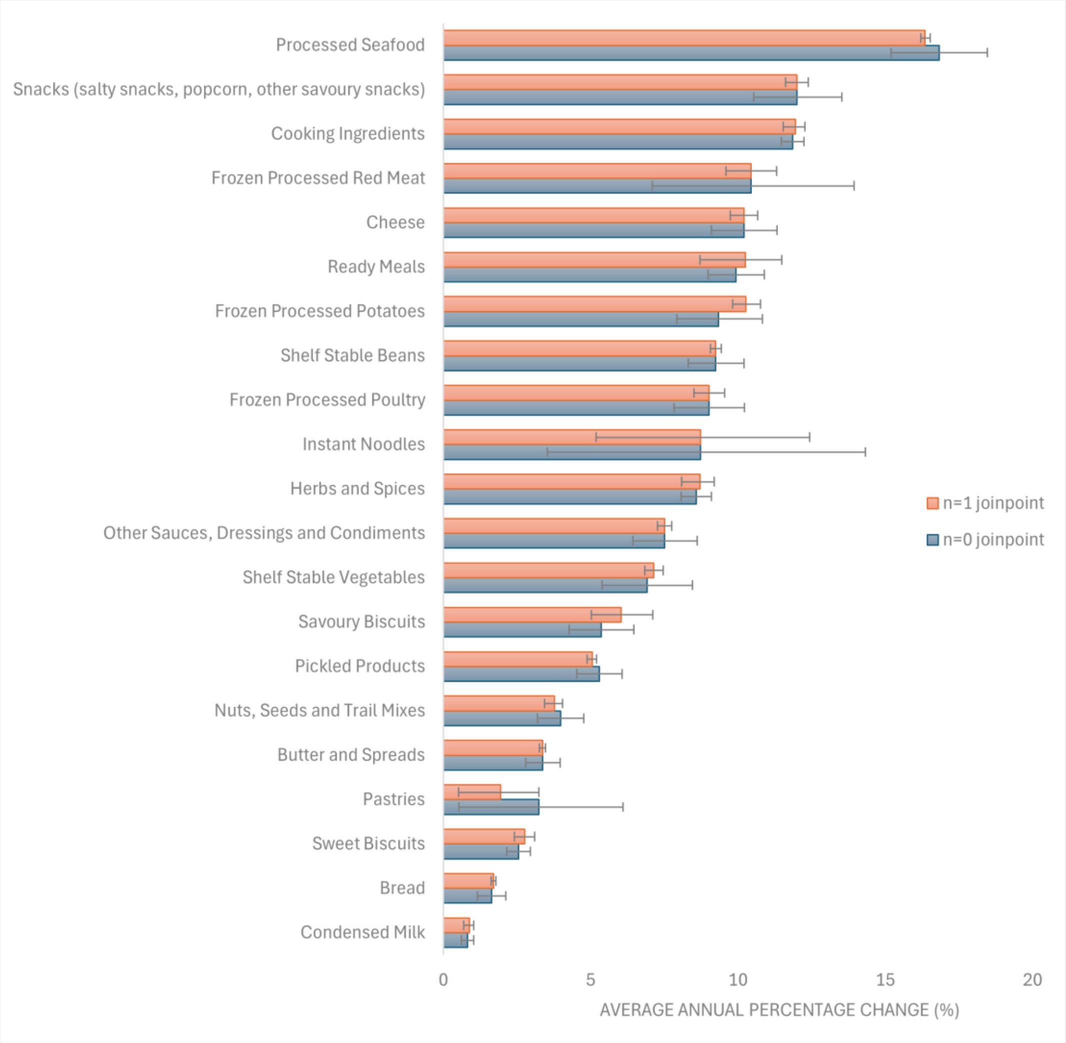

Supplementary File 3 Figure 1. APC estimates using our current method (in blue) compared to average APC estimated using joinpoint (in orange).

Supplementary File 4. Population Health and Cost-Effectiveness Simulation Model (PHACES)

Researchers at Griffith University and The George Institute for Global Health developed Population Health and Cost-Effectiveness Simulation Model (PHACES), a computer model developed to simulate the health impact and cost-effectiveness of dietary changes. The modelling approach has been widely used in evaluating population health impacts and cost-effectiveness of interventions aimed at preventing disease by addressing lifestyle risk factors, including dietary risk factors and physical activity.[26,27]

For the purpose of this study, PHACES includes simulation of sodium intake, systolic blood pressure, and seven cardiovascular or renal diseases (i.e., ischaemic heart disease, ischaemic stroke, intracerebral hemorrhage, subarachnoid hemorrhage, atrial fibrillation and flutter, hypertensive heart disease, and chronic kidney disease).

PHACES is a macro-simulation model with a one-year time-step. For this study the model takes male and female population cohorts in five-year age groups, and simulates annual changes in health and mortality of each cohort until all have died or until a set period has elapsed (i.e., 10 years or 25 years). The model simulated the adult (aged ≥25 years) population alive in the baseline year until all have died. Below this age, the CKD- and CVD-burden related to blood pressure is low and little is known about the blood pressure effects of sodium reduction. The figure below depicts the four distinct health states for each of the diseases (Supplementary File 4, Figure 1). In the model, population health is measured in health-adjusted life years (HALYs) by adding up the years of life that are lived by the population, and then adjusting for time spent in ill-health based on disease-specific disability weights from the Global Burden of Disease (GBD) study.[6]

Probability of dying and size of the health adjustment are influenced by changing rates of incidence, prevalence and mortality of the diseases explicitly simulated in the model. Since PHACES is designed for evaluation of interventions aimed at preventing disease, it simulates the effect of an intervention on incidence (i.e. new cases) of disease. This has flow-on effects on prevalence and mortality from disease as the simulation time progresses, however the model does not explicitly simulate effects of an intervention on case fatality (disease-related mortality) among those who already have disease.

In this model, sodium reduction interventions have an effect on disease incidence via systolic blood pressure. Changes in systolic blood pressure are estimated from the dose-response relationship between dietary sodium intake and systolic blood pressure.[10] PHACES includes relative risks of disease based on meta-analyses undertaken for the GBD study 2019. The relative risks were adjusted for potential confounders in the original cohort studies. These adjusted relative risks were then applied in the Levin-based potential impact fraction calculations to quantify the effects on disease incidences.[11] It should be noted that while there may be some bias with the use of Levin-based potential impact fraction calculations due to risk factor clustering and challenges in confounder adjustment, it is likely to be very modest.[28,29] Alternative methods such as Miettinen's formula, which may have less bias, could not be used because individual study data, often not available in meta-analyses, was required.[29] The potential impact fraction takes the systolic blood pressure distributions (pre- and post-intervention) and the relative risk function for the dose response between systolic blood pressure change and cardiovascular and renal diseases, and combines them with respect to unit blood pressure levels for a given range of plausible values. The potential impact fraction quantifies the proportional change in disease incidence, assuming a theoretical minimum risk exposure level of 115mmHg in which there is minimum or no risk of blood pressure-related events. The change in disease incidence then translates to changes in disease prevalence and subsequently mortality rates, which ultimately propagates to the lifetable where all-cause mortality rates are altered to re-estimate life years.

The model also simulates changes in health care costs, based on the changing rates of disease, the number of people alive, and the unit costs of health care. The unit costs associated with treating modelled diseases are applied per incident and/or prevalent case. Costs associated with all other health care are applied to the number of people alive in the model.

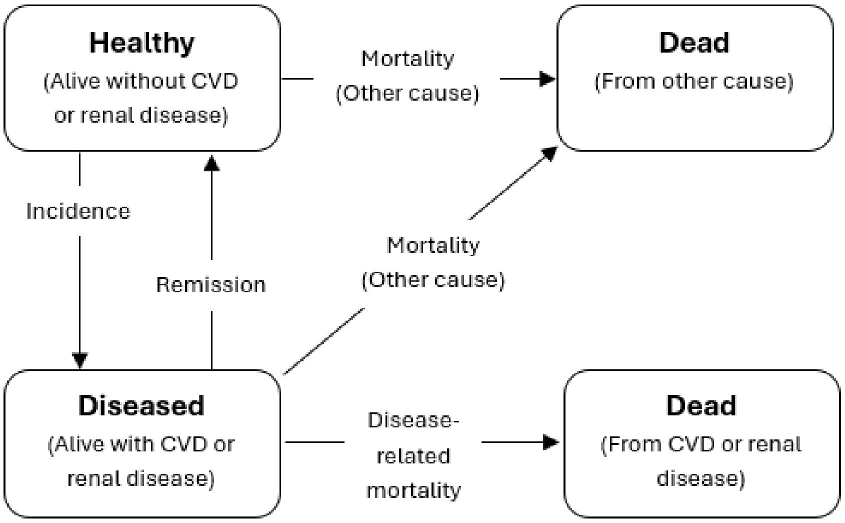

Supplementary File 4 Figure 1: Conceptual model with four states for each disease (adapted from Barendregt et al[30])

Supplementary File 5. Sodium intake, systolic blood pressure and relative risks input data

Supplementary File 5 Table 1. Baseline sodium intake (mg/d), by age and sex.[1-3,31]

Age group (years)	Females	Males
25-29	3482 (701)	3210 (223)
30-34	2724 (334)	2982 (345)
35-39	2751 (209)	3905 (392)
40-44	2729 (193)	3097 (244)
45-49	2996 (250)	2807 (263)
50-54	2375 (179)	4022 (504)
55-59	2261 (89)	2653 (248)
60-64	2060 (135)	3009 (274)
65-69	2523 (276)	3149 (420)
70-74	2508 (263)	3066 (309)
75-79	1471 (259)	3322 (615)
80-84	2326 (275)	2799 (503)
85-89	1689 (213)	2241 (457)
90-94	1689 (213)	2241 (457)
95+	1689 (213)	2241 (457)

Values are mean (standard error)

Supplementary File 5 Table 2. Baseline systolic blood pressure (mmHg), by age and sex.[7,32]

Age group (years)	Females	Males
25-29	114.6 (6.3)	121.3 (6.2)
30-34	116.2 (7.8)	122.4 (7.6)
35-39	119.2 (9.0)	123.5 (8.7)
40-44	122.1 (9.8)	124.8 (9.3)
45-49	124.9 (11.3)	126.6 (10.5)
50-54	130.0 (12.0)	128.1 (10.8)
55-59	133.1 (12.1)	131.6 (11.0)
60-64	136.3 (12.6)	134.1 (11.3)
65-69	138.1 (12.1)	136.2 (10.9)
70-74	140.0 (11.6)	136.6 (10.3)
75-79	141.5 (13.0)	137.6 (11.5)
80-84	142.4 (9.6)	138.7 (8.5)
85-89	141.8 (9.5)	138.2 (8.4)
90-94	140.2 (9.2)	138.1 (8.2)
95+	136.9 (8.7)	136.6 (7.9)

Values are mean (standard deviation).

Supplementary File 5 Table 3. Relative risk estimates and corresponding 95% confidence intervals of cardiovascular disease subtypes and chronic kidney disease per 10 mmHg increase in systolic blood pressure

Risk factor (unit)	Disease	Age interval (y)											
		**25-29**	**30-34**	**35-39**	**40-44**	**45-49**	**50-54**	**55-59**	**60-64**	**65-69**	**70-74**	**75-79**	**80+**
**Systolic blood pressure (per 10 mmHg)**	Ischemic heart disease	1.97 (1.44-2.60)	1.82 (1.46-2.21)	1.67 (1.46-1.91)	1.57 (1.40-1.80)	1.53 (1.39-1.71)	1.49 (1.39-1.62)	1.45 (1.37-1.54)	1.41 (1.33-1.49)	1.36 (1.26-1.46)	1.33 (1.22-1.42)	1.30 (1.23-1.40)	1.27 (1.13-1.44)
	Ischemic stroke	1.85 (1.40-2.59)	1.77 (1.43-2.25)	1.69 (1.40-2.04)	1.63 (1.35-1.95)	1.57 (1.36-1.82)	1.52 (1.36-1.70)	1.47 (1.34-1.60)	1.41 (1.30-1.52)	1.36 (1.21-1.49)	1.32 (1.17-1.45)	1.28 (1.18-1.39)	1.20 (1.11-1.37)
	Intracerebral hemorrhage	2.13 (1.56-2.92)	2.05 (1.59-2.65)	1.97 (1.59-2.47)	1.87 (1.49-2.30)	1.78 (1.48-2.11)	1.68 (1.45-1.93)	1.58 (1.40-1.75)	1.48 (1.33-1.62)	1.38 (1.21-1.54)	1.32 (1.16-1.50)	1.31 (1.19-1.45)	1.28 (1.13-1.52)
	Subarachnoid hemorrhage	2.13 (1.56-2.92)	2.05 (1.59-2.65)	1.97 (1.59-2.47)	1.87 (1.49-2.30)	1.78 (1.48-2.11)	1.68 (1.45-1.93)	1.58 (1.40-1.75)	1.48 (1.33-1.62)	1.38 (1.21-1.54)	1.32 (1.16-1.50)	1.31 (1.19-1.45)	1.28 (1.13-1.52)
	Hypertensive heart disease	2.86 (1.83-4.11)	2.84 (1.86-4.19)	2.81 (1.80-4.34)	2.70 (1.76-4.19)	2.50 (1.80-3.76)	2.30 (1.77-3.46)	2.11 (1.65-3.34)	1.91 (1.45-3.17)	1.71 (1.19-3.22)	1.62 (1.05-3.14)	1.64 (1.08-3.09)	1.71 (1.10-3.26)
	Atrial fibrillation and flutter	1.76 (1.34-2.43)	1.63 (1.38-2.03)	1.50 (1.40-1.64)	1.42 (1.34-1.51)	1.39 (1.33-1.46)	1.36 (1.31-1.41)	1.33 (1.29-1.37)	1.30 (1.27-1.33)	1.27 (1.23-1.31)	1.24 (1.20-1.28)	1.21 (1.18-1.24)	1.13 (1.09-1.19)
	Chronic kidney disease[‖]	1.28 (1.18-1.39)	1.28 (1.18-1.39)	1.28 (1.18-1.39)	1.28 (1.18-1.39)	1.28 (1.18-1.39)	1.28 (1.18-1.39)	1.28 (1.18-1.39)	1.28 (1.18-1.39)	1.28 (1.18-1.39)	1.28 (1.18-1.39)	1.28 (1.18-1.39)	1.28 (1.18-1.39)

Supplementary File 6. Impact of reformulation in compliance with WHO global benchmarks* on sodium intake by sex over 30 years^

*We assumed the WHO global sodium benchmark levels were achieved after a 4-year implementation period and then sodium content in foods remained the same thereafter.

^Sodium intake from packaged foods was projected from 2014 data for 2019 and onwards, accounting for increasing consumption of packaged foods over time until sodium contributed from packaged foods reached 50% of total sodium intake.

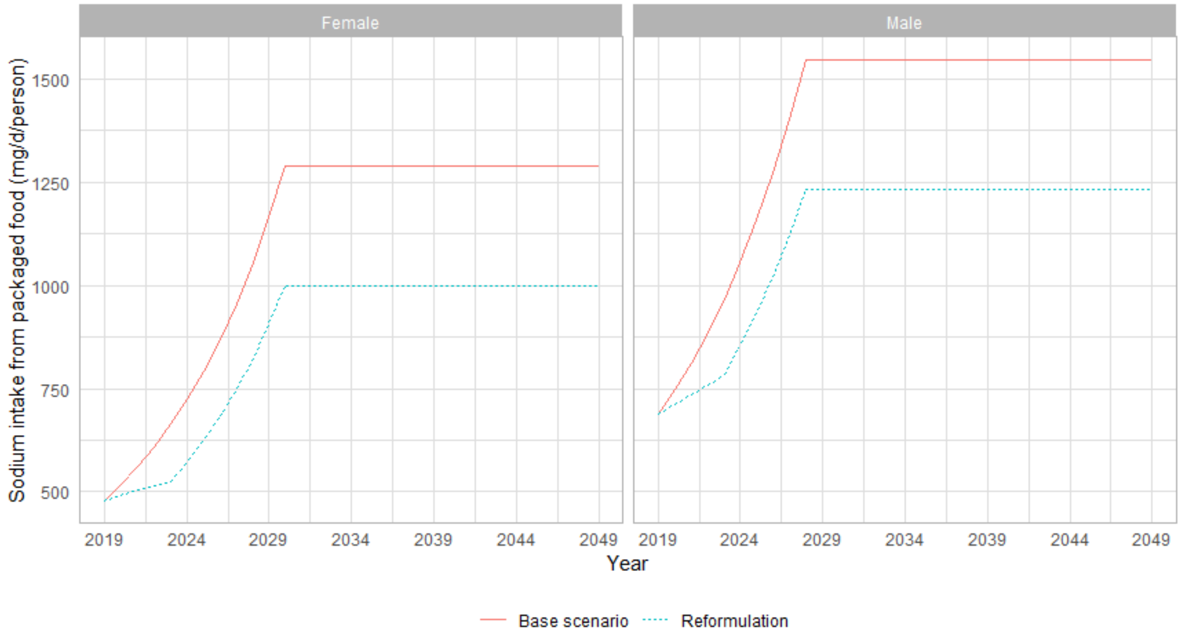

Supplementary File 7. Annual percentage change (%) of consumption for different packaged food groups from 2011 to 2021*

Dietary food groups	Euromonitor food group	WHO benchmarking group	Annual percentage change (%) (95% CI)	P-value
Snacks	Snacks (‘Salty snacks’, ‘Popcorn’, and ‘Other savoury snacks’)	Snacks (‘Potato, vegetable and grain chips’, ‘Extruded snacks’)	12.0 (11.0-13.0)	<0.0001
Bread	Bread	Leavened bread	1.6 (1.4-1.9)	<0.0001
Cereal-based products	Instant noodles	Pasta, noodles, and rice or grains with sauce or seasoned (dry-mix, concentrated)	8.7 (4.5-13.1)	0.0024
Cereal grain	Ready meals	Ready-to-eat meals composed of a combination of carbohydrate and either vegetable or meat, or all three combined	9.9 (9.2-10.6)	<0.0001
Cheese	Cheese	Cheese (‘Fresh unripened cheese’, ‘Semi-hard ripened cheese’, ‘Processed cheese’)	10.2 (9.3-11.1)	<0.0001
Dried salted fish	Processed seafood	Processed fish and seafood products, non- heat-treated	16.8 (15.8-17.8)	<0.0001
Fats, edible oils	Butter and spreads	Salted butter, butter blends, margarine and oil-based spreads	3.4 (2.9-3.8)	<0.0001
Herbs, spices	Herbs and spices	Marinades and thick pastes	8.6 (8.1-9.1)	<0.0001
Noodles	Instant noodles	Pasta, noodles, and rice or grains with sauce or seasoned (dry-mix, concentrated)	8.7 (4.5-13.1)	0.0024
Nuts and seeds	Nuts, seeds and trail mixes	Nuts, seeds and kernels	4.0 (3.5-4.4)	<0.0001
Other vegetables	Shelf stable vegetables	Canned vegetables and legumes	6.9 (5.9-7.9)	<0.0001
Pastry	Pastries	Pies and pastries	3.2 (1.3-5.2)	0.0089
Poultry	Frozen processed poultry	Comminuted meat products, heat treated (cooked)	9.0 (8.0-10.0)	<0.0001
Pulses and legumes	Shelf stable beans	Canned vegetables and legumes	9.3 (8.7-9.8)	<0.0001
Roots tubers	Frozen processed potatoes	Frozen potatoes and other potato products (ready-to-eat)	9.3 (8.0-10.6)	<0.0001
Beef	Frozen processed red meat	Raw meat products and preparations	10.4 (8.4-12.5)	<0.0001
Buffalo	Frozen processed red meat	Raw meat products and preparations	10.4 (8.4-12.5)	<0.0001
Chutney	Other sauces,dips and condiments	Dips and dipping sauces	7.5 (6.9-8.2)	<0.0001
Crab	Processed seafood	Processed fish and seafood products, raw	16.8 (15.8-17.8)	<0.0001
Custard	Condensed milk	Baked and cooked desserts	0.8 (0.7-0.9)	<0.0001
Goat	Frozen processed red meat	Raw meat products and preparations	10.4 (8.4-12.5)	<0.0001
Mutton	Frozen processed red meat	Raw meat products and preparations	10.4 (8.4-12.5)	<0.0001
Pickles	Pickled products	Pickled vegetables	5.3 (4.8-5.8)	<0.0001
Prawn	Processed seafood	Processed fish and seafood products, raw	16.8 (15.8-17.8)	<0.0001
Sauces	Cooking ingredients	Cooking sauces including pasta sauces and tomato sauces (not concentrated)	11.9 (11.5-12.2)	<0.0001
Sausages	Frozen processed red meat	Raw meat products and preparations	10.4 (8.4-12.5)	<0.0001
Savoury biscuits	Savoury biscuits	Crackers/savoury biscuits	5.4 (4.4-6.4)	<0.0001
Sweet biscuits	Sweet biscuits	Cookies/sweet biscuits	2.5 (2.2-2.9)	<0.0001

* This annual increase rate was estimated using Euromonitor sales data from 2011 to 2021

Supplementary File 8. Top ten largest sodium reduction (mg/day) post-reformulation at Year 4 by WHO global sodium benchmark food categories

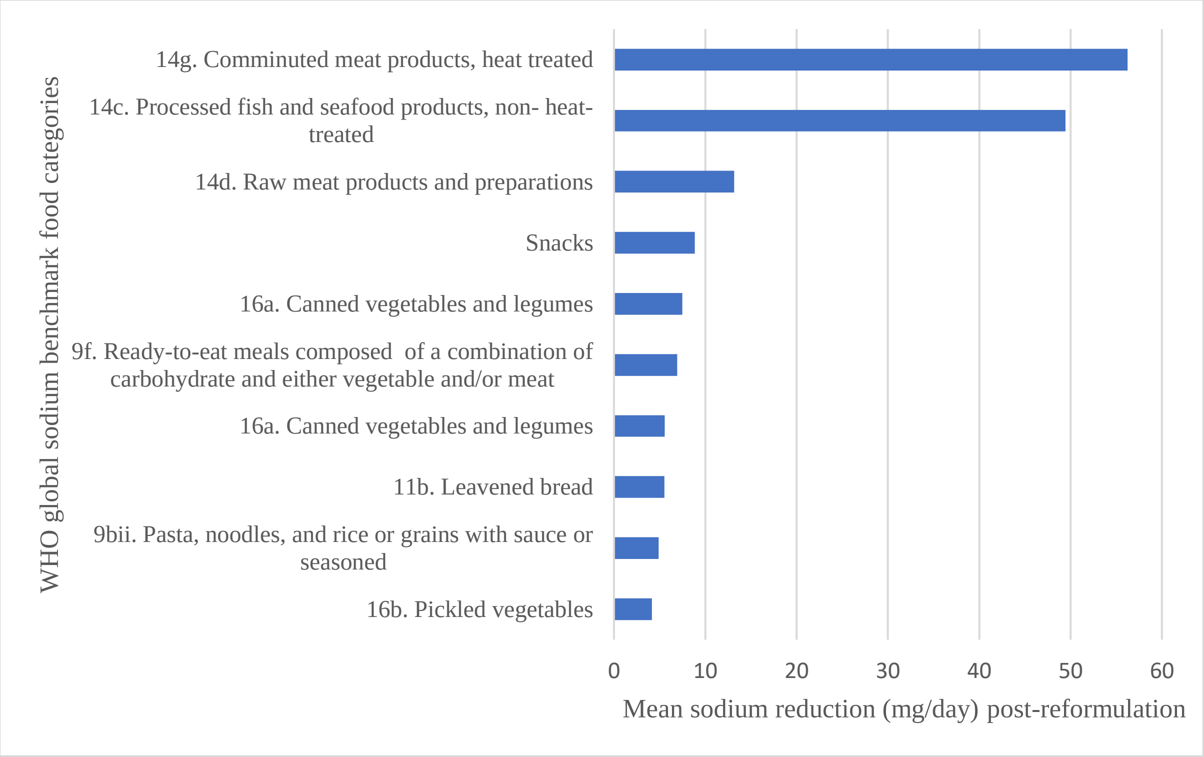

Supplementary File 9. Mean sodium levels in packaged foods in India compared against WHO global sodium benchmarks

WHO Category*	Number of products	Median (IQR) sodium level (mg/100g)	WHO sodium benchmark (mg/100g)	Number (%) of products with sodium level above WHO targets
2a. Cookies/sweet biscuits	205	200 (125, 354)	265	75 (37)
2b. Cakes and sponges	30	312 (158, 427)	205	19 (63)
2c. Pies and pastries	3	327 (317, 353)	120	3 (100)
2d. Baked and cooked desserts	23	40 (18, 83)	100	6 (26)
3a. Crackers/savoury biscuits	62	430 (275, 662)	600	19 (31)
3b. Nuts, seeds and kernels	171	411 (217, 734)	280	119 (70)
3c. Potato, vegetable and grain chips	214	640 (480, 827)	500	154 (72)
3d. Extruded snacks	373	672 (433, 890)	520	242 (65)
3e. Pretzels	1	1122 (NA)	760	1 (100)
6a. Minimally processed breakfast cereals (includes all types – prepared, ready-made and dry-mixes)	14	117 (30, 418)	100	8 (57)
6b. Highly processed breakfast cereals	174	369 (248, 650)	280	123 (71)
8a. Fresh unripened cheese	21	1000 (560, 1000)	190	21 (100)
8b. Soft to medium ripened cheese	9	670 (669, 1000)	520	7 (78)
8c. Semi-hard ripened cheese	13	804 (700, 1000)	625	12 (92)
8e. Mould ripened cheese, white and red	1	790 (NA)	510	1 (100)
8g. Processed cheese	62	1252 (1000, 1426)	720	55 (89)
9a. Canned foods	20	294 (214, 336)	225	14 (70)
9bi. Pasta, noodles, and rice or grains with sauce or seasoned (prepared)	11	390 (160, 400)	230	7 (64)
9bii. Pasta, noodles, and rice or grains with sauce or seasoned (dry-mix, concentrated)	197	1232 (790, 1530)	770	150 (76)

**WHO Category***	**Number of products**	**Median (IQR) sodium level (mg/100g)**	**WHO sodium benchmark (mg/100g)**	**Number (%) of products with sodium level above WHO targets**
9c. Pizza and pizza snacks	7	381 (259, 431)	450	2 (29)
9d. Sandwiches and wraps	1	591 (NA)	430	1 (100)
9f. Ready-to-eat meals composed of a combination of carbohydrate and either vegetable or meat, or all three combined	59	480 (392, 578)	250	58 (98)
9gi. Soups (ready-to- serve, canned and refrigerated soups)	14	233 (202, 250)	235	7 (50)
9gii. Soups (dry soup only) (concentrated)	103	4194 (1117, 4817)	1,200	77 (75)
10a. Salted butter, butter blends, margarine and oil-based spreads	37	786 (750, 836)	400	35 (95)
11a. Sweet and raisin breads	2	330 (297, 362)	310	1 (50)
11b. Leavened bread	18	476 (136, 517)	330	12 (67)
11c. Flatbreads	81	990 (407, 1984)	320	67 (83)
14a. Canned fish	32	415 (154, 485)	360	19 (59)
14b. Processed fish and seafood products, raw	22	439 (375, 630)	270	18 (82)
14c. Processed fish and seafood products, non- heat-treated	11	440 (323, 1925)	800	4 (36)
14d. Raw meat products and preparations	4	567 (531, 595)	230	4 (100)
14ei. Whole muscle meat products, heat treated (frozen and canned products)	3	500 (384, 617)	270	2 (67)
14eii. Whole muscle meat products, heat treated (refrigerated products)	4	678 (647, 730)	600	3 (75)
14g. Comminuted meat products, heat treated (cooked)	8	590 (491, 787)	540	5 (62)
14h. Comminuted meat products, non-heat preservation	2	447 (341, 552)	830	0 (0)
16a. Canned vegetables and legumes	37	29 (12, 272)	50	14 (38)
16b. Pickled vegetables	89	3383 (820, 4900)	550	78 (88)

WHO Category*	Number of products	Median (IQR) sodium level (mg/100g)	WHO sodium benchmark (mg/100g)	Number (%) of products with sodium level above WHO targets
16c. Olives and sundried tomatoes	44	1002 (675, 1594)	780	32 (73)
16f. Frozen potatoes and other potato products (ready-to-eat)	5	374 (300, 446)	260	4 (80)
18ai. Bouillon and soup stock (not concentrated)	1	395 (NA)	350	1 (100)
18aii. Bouillon and soup stock (concentrated)	4	5140 (4622, 10396)	15,000	1 (25)
18b. Cooking sauces including pasta sauces and tomato sauces (not concentrated)	83	384 (361, 400)	330	77 (93)
18c. Dips and dipping sauces	30	744 (437, 2388)	360	25 (83)
18d. Emulsion-based dips, sauces and dressings	59	701 (500, 837)	500	43 (73)
18e. Condiments	167	1007 (775, 1200)	650	145 (87)
18f. Soy sauce and fish sauce	19	6800 (6300, 7762)	4,840	17 (89)
18g. Other Asian-style sauces	23	2490 (1204, 4163)	680	21 (91)
18h. Marinades and thick pastes	152	4269 (2000, 8000)	1,425	129 (85)

*WHO global sodium benchmarks relate to food categories that are major sources of sodium globally, and thus the benchmarks and food categories may not always capture proprietary and traditional foods commonly consumed in India.

Supplementary file 10. Annually updated cumulative estimates of averted new cases of total cardiovascular diseases* (CVD) (A), ischemic heart disease (IHD) (B), stroke (C), atrial fibrillation (Afib) (D), hypertensive heart disease (HHD) (E), and chronic kidney disease (CKD) (F), if the WHO global sodium benchmarks for packaged foods were achieved in India. The line represents the mean and shaded area shows the 95% uncertainty intervals.

*Total CVD includes ischemic heart disease, ischemic stroke, intracerebral and subarachnoid hemorrhage, atrial fibrillation and flutter, and hypertensive heart disease.

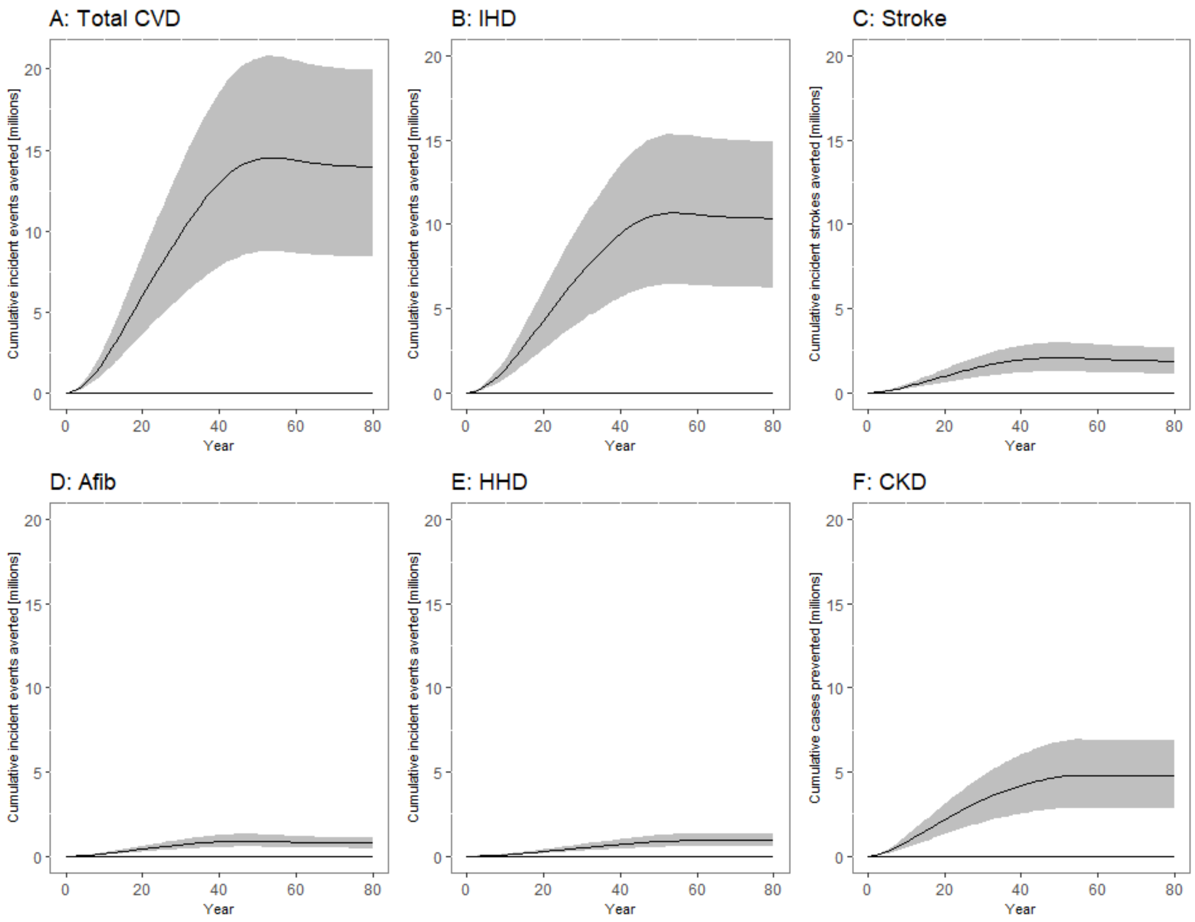

Supplementary File 11. Annually updated cumulative estimates of averted deaths from total cardiovascular diseases (A), ischemic heart disease (B), stroke (C), atrial fibrillation (D), hypertensive heart disease (E), and chronic kidney disease (F), if the WHO global sodium benchmarks for packaged foods were achieved in India. The line represents the mean and shaded area shows the 95% uncertainty intervals.

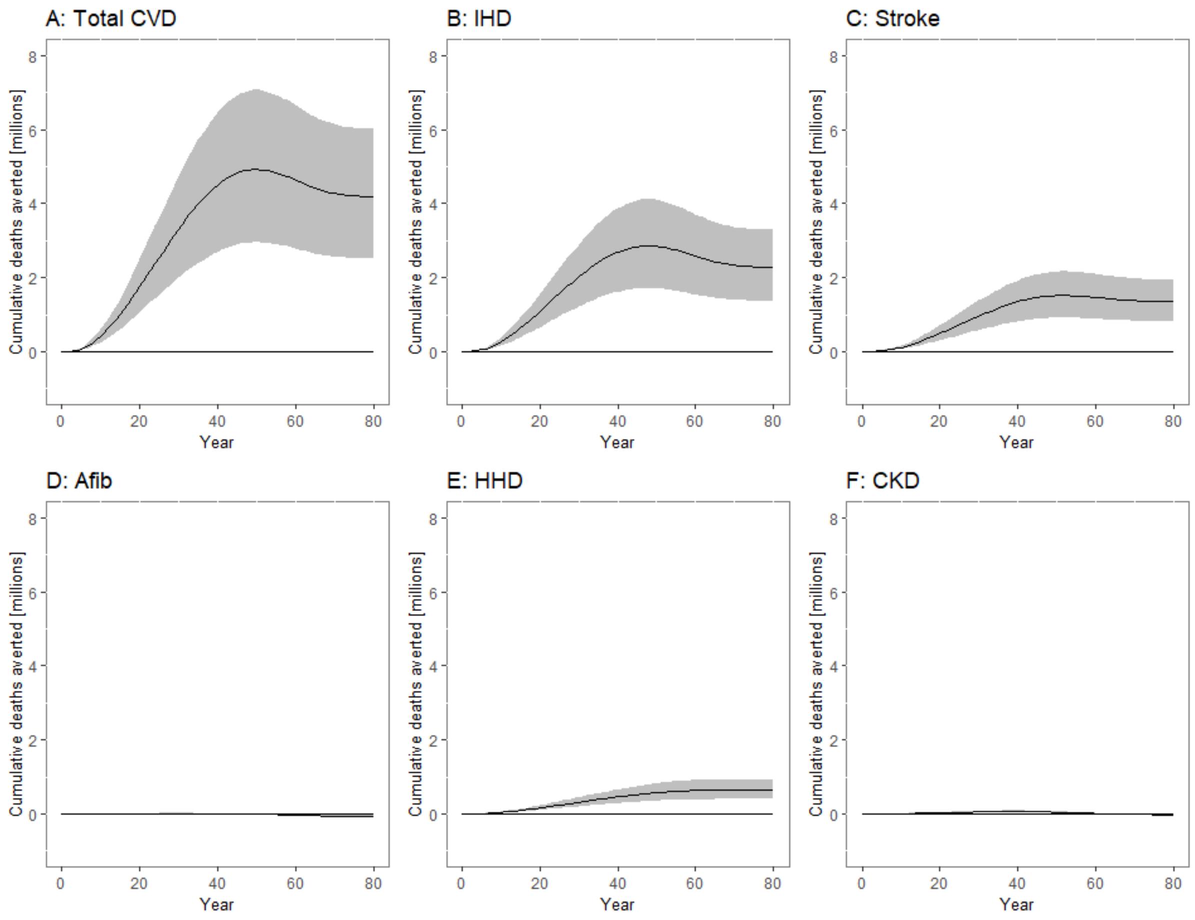

Supplementary File 12. Annually updated cumulative estimates of health-adjusted life years (HALYs) gained in A) the total adult Indian population, and B) separately in women (red) and men (blue), if the WHO global sodium benchmarks for packaged foods were achieved in India. The line represents the mean and shaded area shows the 95% uncertainty intervals. The observed higher estimated health gains accrued among men compared to women is due to higher sodium intake from packaged foods and greater CVD burden in men compared to women.

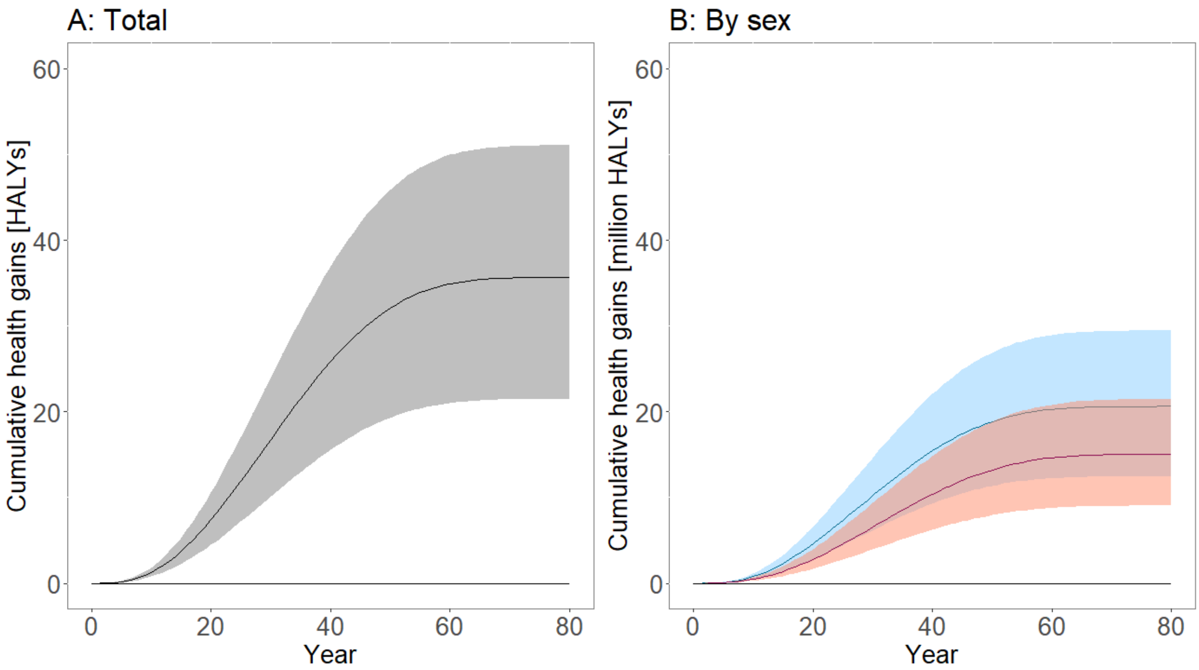

Supplementary File 13. Government intervention costs of legislating and monitoring the WHO global sodium benchmarks in India based on 2019 costs (Indian rupees)

		Partial implementation (years 1-3)			Full implementation (year 4)
**HUMAN RESOURCES (incl. consultants)**	***Roles / responsibilities***				
Program management (incl. monitoring & evaluation-M & E)		75,875,127	75,875,127	75,875,127	75,875,127
Promotion / media / advocacy		73,878,317	73,878,317	73,878,317	73,878,317
Law enforcement / inspection		129,112,701	129,112,701	129,112,701	129,112,701
National-level technical assistance by international consultant	*(local planning / implementation)*	192,043	192,043	192,043	192,043
**TOTAL HUMAN RESOURCES**		279,058,189	279,058,189	279,058,189	279,058,189
**TRAINING (for programme staff)**	***Purpose***				
Training workshop (1)	*(salt and public health)*	90,280,136	90,280,136	90,280,136	90,304,452
Training workshop (2)	*(food inspection)*	45,010,669	45,010,669	45,010,669	45,010,669
**TOTAL TRAINING**		135,290,805	135,290,805	135,290,805	135,315,121
**MEETINGS (involving external agencies)**	***Purpose***				
Meetings / workshops (1)	*(planning, + M&E)*	32,198,747	32,198,747	32,198,747	16,782,694
**TOTAL MEETINGS**		32,198,747	32,198,747	32,198,747	16,782,694
**MASS MEDIA**	***Campaign description***				
Television, radio, newspapers, flyers/ leaflets	*Half at national, half at provincial level*	1,188,018,749	1,188,018,749	1,188,018,749	1,188,018,749
**TOTAL MEDIA**		1,188,018,749	1,188,018,749	1,188,018,749	1,188,018,749
**SUPPLIES & EQUIPMENT**	***Purpose / need***				
**TOTAL SUPPLIES & EQUIPMENT**	*Fuel for vehicles, computer, printer, photocopier, car*	166,628,850	6,732,717	6,732,717	6,732,717
**TOTAL**		**1,801,195,338**	**1,641,299,203**	**1,641,299,203**	**1,625,907,467**

In the absence of actual intervention cost data (given India has not implemented such an intervention), the cost estimates are derived from the WHO NCD Costing Tool which provides the estimates of the cost of a national sodium reduction intervention in India using an ingredients-based (bottom up) approach. Only government intervention costs were included because food products are often reformulated as part of the natural product cycle and therefore associated reformulation costs are absorbed within the food manufacturer’s standard running costs. Resources required to deliver the sodium reduction intervention are identified, quantified and costed. Given the substantial healthcare cost savings of implementing the WHO global sodium benchmarks (shown in Table 3), deviations in the intervention costs are unlikely to affect the findings.

Supplementary File 14. Health-adjusted life years (HALYs) gained, net costs, and incremental cost-effectiveness ratios estimated over the first 10 years, 25 years and population lifetime in the main analysis and deterministic sensitivity analyses

	Mean (95% Uncertainty Interval)			Probability	
Analysis	HALYs gained (millions)	Net cost (USD billion)	Incremental cost-effectiveness ratio (USD/HALY)	Cost-saving	Cost-effective
Main analysis over the first 10 years	0.98 (0.59; 1.43)	-0.80 (-1.44; -0.34)	dominant (dominant; dominant)	100%	100%
Sensitivity analyses over the first 10 years					
10-year implementation time	0.53 (0.32; 0.77)	-0.35 (-0.76; -0.05)	dominant (dominant; dominant)	99.5%	100%
Packaged food consumption increases according to APC until contributing to 80% of total sodium intake	0.99 (0.60; 1.43)	-0.81 (-1.46; -0.34)	dominant (dominant; dominant)	100%	100%
Intake of packaged foods increases linearly until contributing to 50% of total sodium intake	1.00 (0.60; 1.45)	-0.77 (-1.41; -0.33)	dominant (dominant; dominant)	100%	100%
Intake of packaged foods does not increase from baseline	0.63 (0.38; 0.92)	-0.42 (-0.80; -0.15)	dominant (dominant; dominant)	100%	100%
0% discounting of HALYs and costs	1.21 (0.73; 1.77)	-0.98 (-1.77; -0.42)	dominant (dominant; dominant)	100%	100%
5% discounting of HALYs and costs	0.85 (0.52; 1.24)	-0.69 (-1.26; -0.29)	dominant (dominant; dominant)	100%	100%
Main analysis over the first 25 years	11.1 (6.4; 16.0)	-3.3 (-6.4; -1.2)	dominant (dominant; dominant)	100%	100%
Sensitivity analyses over the first 25 years					
10-year implementation time	9.3 (5.3; 13.4)	-2.9 (-5.7; -1.0)	dominant (dominant; dominant)	100%	100%
Packaged food consumption increases according to APC until contributing to 80% of total sodium intake	13.6 (7.9; 19.6)	-4.6 (-8.7; -1.7)	dominant (dominant; dominant)	100%	100%
Intake of packaged foods increases linearly until contributing to 50% of total sodium intake	10.4 (6.0; 15.0)	-3.1 (-6.0; -1.1)	dominant (dominant; dominant)	100%	100%
Intake of packaged foods does not increase from baseline	5.1 (3.0; 7.4)	-1.3 (-2.7; -0.4)	dominant (dominant; dominant)	99.8%	100%
0% discounting of HALYs and costs	18.8 (10.8; 27.1)	-5.1 (-10.2; -1.7)	dominant (dominant; dominant)	99.9%	100%
5% discounting of HALYs and costs	7.9 (4.5; 11.4)	-2.5 (-4.8; -0.9)	dominant (dominant; dominant)	100%	100%
Main analysis over the population lifetime	35.9 (20.9; 51.3)	-2.5 (-8.2; 2.7)	dominant (dominant; 71.4)	84.2%	100%
Sensitivity analyses over the population lifetime					
10-year implementation time	33.5 (19.5; 47.9)	-2.2 (-7.6; 2.7)	dominant (dominant; 76.1)	83.0%	100%
Packaged food consumption increases according to APC until contributing to 80% of total sodium intake	52.7 (30.8; 75.1)	-3.6 (-12.1; 4.2)	dominant (dominant; 75.0)	83.8%	100%
Intake of packaged foods increases linearly until contributing to 50% of total sodium intake	34.2 (19.9; 48.9)	-2.3 (-7.8; 2.7)	dominant (dominant; 75.4)	83.7%	100%
Intake of packaged foods does not increase from baseline	14.3 (8.3; 20.6)	-0.9 (-3.1; 1.2)	dominant (dominant; 80.2)	80.9%	100%
0% discounting of HALYs and costs	103.0 (60.0; 147.2)	0.0 (-14.3; 15.4)	dominant (dominant; 143.1)	51.7%	100%
5% discounting of HALYs and costs	19.5 (11.3; 27.8)	-2.3 (-5.8; 0.5)	dominant (dominant; 27.7)	94.3%	100%

APC, Annual percentage change; HALYs, health-adjusted life years

References

1. Johnson C, Santos JA, Sparks E, et al. Sources of Dietary Salt in North and South India Estimated from 24 Hour Dietary Recall. *Nutrients*. 2019;11(2):318.
2. Gopalan CR, B.; Balasubramanian, S.; Rao Narasinga, B.; Deosthale, Y.; Pant, K. Nutritive Values of Indian Foods. National Institute of Nutrition. https://www.scribd.com/document/383179301/Nutritive-values-Indian-food-pdf#
3. Euromonitor International. Passport Processed Foods and Beverages. Euromonitor International. https://www.euromonitor.com/insights/food-and-nutrition
4. Dunford EK, Neal B. FoodSwitch and use of crowdsourcing to inform nutrient databases. *J Food Compost Anal*. 2017/12/01/ 2017;64:13-17. doi:https://doi.org/10.1016/j.jfca.2017.07.022
5. Global Burden of Disease Collaborative Network. *Global Burden of Disease Study 2019 (GBD 2019) Population Estimates 1950-2019*. 2020. http://ghdx.healthdata.org/record/ihme-data/gbd-2019-population-estimates-1950-2019
6. Institute for Health Metrics and Evaluation (IHME). GBD Results Tool. Accessed May 10, 2021. http://ghdx.healthdata.org/gbd-results-tool
7. Ramakrishnan S, Zachariah G, Gupta K, et al. Prevalence of hypertension among Indian adults: Results from the great India blood pressure survey. *Indian Heart Journal*. 2019/07/01/ 2019;71(4):309-313. doi:https://doi.org/10.1016/j.ihj.2019.09.012
8. Forouzanfar MH, Liu P, Roth GA, et al. Global Burden of Hypertension and Systolic Blood Pressure of at Least 110 to 115 mm Hg, 1990-2015. *JAMA*. Jan 10 2017;317(2):165-182. doi:10.1001/jama.2016.19043
9. GBD Chronic Kidney Disease Collaboration. Global, regional, and national burden of chronic kidney disease, 1990-2017: a systematic analysis for the Global Burden of Disease Study 2017. *The Lancet*. 2020;395(10225):709-733. doi:10.1016/S0140-6736(20)30045-3
10. Filippini T, Malavolti M, Whelton PK, Naska A, Orsini N, Vinceti M. Blood Pressure Effects of Sodium Reduction. *Circulation*. 2021;143(16):1542-1567. doi:doi:10.1161/CIRCULATIONAHA.120.050371
11. Barendregt JJ, Van Oortmarssen GJ, Vos T, Murray CJ. A generic model for the assessment of disease epidemiology: the computational basis of DisMod II. *Popul Health Metr*. Apr 14 2003;1(1):4. doi:10.1186/1478-7954-1-4
12. Gummidi B, John O, John R, et al. Catastrophic Health Expenditure and Distress Financing Among Patients With Nondialysis Chronic Kidney Disease in Uddanam, India. *Kidney International Reports*. 2022/02/01/ 2022;7(2):319-321. doi:https://doi.org/10.1016/j.ekir.2021.10.015
13. Shaikh M, Woodward M, John O, et al. Utilization, costs, and outcomes for patients receiving publicly funded hemodialysis in India. *Kidney international*. 2018/09/01/ 2018;94(3):440-445. doi:https://doi.org/10.1016/j.kint.2018.03.028
14. Bikbov B, Purcell CA, Levey AS, et al. Global, regional, and national burden of chronic kidney disease, 1990–2017: a systematic analysis for the Global Burden of Disease Study 2017. *The Lancet*. 2020;395(10225):709-733. doi:10.1016/S0140-6736(20)30045-3
15. Das H, Moran AE, Pathni AK, Sharma B, Kunwar A, Deo S. Cost-Effectiveness of Improved Hypertension Management in India through Increased Treatment Coverage and Adherence: A Mathematical Modeling Study. *Glob Heart*. May 10 2021;16(1):37. doi:10.5334/gh.952
16. Rajasulochana SR, Kar SS. Economic burden associated with stroke in India: insights from national sample survey 2017-18. *Expert Review of Pharmacoeconomics & Outcomes Research*. 2022/04/03 2022;22(3):455-463. doi:10.1080/14737167.2021.1941883
17. Stevens B, Pezzullo L, Verdian L, Tomlinson J, George A, Bacal F. The Economic Burden of Heart Conditions in Brazil. *Arquivos brasileiros de cardiologia*. Jul 2018;111(1):29-36. doi:10.5935/abc.20180104
18. Malhotra S, Karan R, Pandhi P, Jain S. Pattern of use and pharmacoeconomic impact of antihypertensive drugs in a north Indian referral hospital. *European Journal of Clinical Pharmacology*. 2001/09/01 2001;57(6):535-539. doi:10.1007/s002280100333
19. Dhillon P, Ladusingh L. Implications of Age Profile of Healthcare Expenditure and Age Structural Transition on Future Healthcare Spending in India https://paa2014.populationassociation.org/papers/141816
20. National Health Accounts Technical Secretariat NHSRC, Ministry of Health and Family Welfare, Government of India. National Health Accounts Estimates for India 2018-19. Ministry of Health and

Family Welfare, Government of India. Accessed 12 July, 2023.
https://nhsrcindia.org/sites/default/files/2022-09/NHA%202018-19_07-09-2022_revised_0.pdf
21. World Health Organization. Scaling up action against non-communicable diseases: how much will it cost? World Health Organization,.
https://apps.who.int/iris/bitstream/handle/10665/44706/9789241502313_eng.pdf
22. Smith LC. The great Indian calorie debate: Explaining rising undernourishment during India's rapid economic growth. *Food Policy*. 2015/01/01/ 2015;50:53-67. doi:https://doi.org/10.1016/j.foodpol.2014.10.011
23. Landais E, Miotto-Plessis M, Bene C, et al. Consumption of food away from home in low- and middle-income countries: a systematic scoping review. *Nutrition Reviews*. 2022;doi:10.1093/nutrit/nuac085
24. Bhat S, Marklund M, Henry ME, et al. A Systematic Review of the Sources of Dietary Salt Around the World. *Advances in Nutrition*. 2020;11(3):677-686. doi:10.1093/advances/nmz134 %J Advances in Nutrition
25. Clegg LX, Hankey BF, Tiwari R, Feuer EJ, Edwards BK. Estimating average annual per cent change in trend analysis. *Statistics in medicine*. 2009;28(29):3670-3682.
26. Marklund M, Zheng M, Veerman JL, Wu JHY. Estimated health benefits, costs, and cost-effectiveness of eliminating industrial trans-fatty acids in Australia: A modelling study. *PLOS Medicine*. 2020;17(11):e1003407. doi:10.1371/journal.pmed.1003407
27. Aminde LN, Cobiac L, Veerman JL. Cost-effectiveness analysis of population salt reduction interventions to prevent cardiovascular disease in Cameroon: mathematical modelling study. 2020;10(11):e041346. doi:10.1136/bmjopen-2020-041346 %J BMJ Open
28. Ferguson J, Alvarez A, Mulligan M, Judge C, O'Donnell M. Bias assessment and correction for Levin's population attributable fraction in the presence of confounding. *Eur J Epidemiol*. Feb 2024;39(2):111-119. doi:10.1007/s10654-023-01063-8
29. Khosravi A, Nielsen RO, Mansournia MA. Methods matter: population attributable fraction (PAF) in sport and exercise medicine. *Br J Sports Med*. Sep 2020;54(17):1049-1054. doi:10.1136/bjsports-2020-101977
30. Barendregt JJ, van Oortmarssen GJ, Vos T, Murray CJL. A generic model for the assessment of disease epidemiology: the computational basis of DisMod II. *Population Health Metrics*. 2003/04/14 2003;1(1):4. doi:10.1186/1478-7954-1-4
31. Dunford E, Webster J, Metzler AB, et al. International collaborative project to compare and monitor the nutritional composition of processed foods. *European Journal of Preventive Cardiology*. 2012/12/01 2011;19(6):1326-1332. doi:10.1177/1741826711425777
32. GBD Risk Factors Collaborators. Global burden of 87 risk factors in 204 countries and territories, 1990-2019: a systematic analysis for the Global Burden of Disease Study 2019. *Lancet*. 2020;396(10258):1223-1249. doi:10.1016/S0140-6736(20)30752-2
